# Carbon Capture: Theoretical Guidelines for Activated Carbon-Based CO_2_ Adsorption Material Evaluation

**DOI:** 10.1021/acs.jpclett.3c02711

**Published:** 2023-11-21

**Authors:** Drew M. Glenna, Asmita Jana, Qiang Xu, Yixiao Wang, Yuqing Meng, Yingchao Yang, Manish Neupane, Lucun Wang, Haiyan Zhao, Jin Qian, Seth W. Snyder

**Affiliations:** † Department of Nuclear Engineering & Industrial Management, University of Idaho, Idaho Falls, Idaho 83402, United States; ‡ Chemical Sciences Division, Lawrence Berkeley National Laboratory, Berkeley, California 94720, United States; § Advanced Light Source, Lawrence Berkeley National Laboratory, Berkeley, California 94720, United States; ∥ Energy & Environmental Science and Technology, Idaho National Laboratory, Idaho Falls, Idaho 83415, United States; # Department of Mechanical Engineering, University of Maine, Orono, Maine 04469, United States; ∇ Department of Chemical and Biological Engineering, University of Idaho, Idaho Falls, Idaho 83402, United States

## Abstract

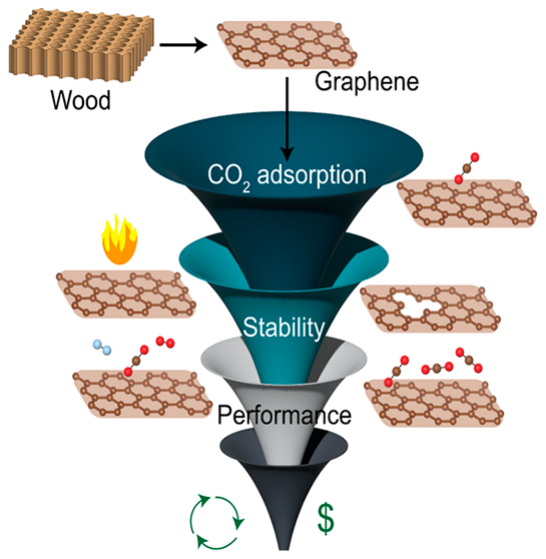

Activated carbon (AC)-based materials have shown promising performance in carbon capture, offering low cost and sustainable sourcing from abundant natural resources. Despite ACs growing as a new class of materials, theoretical guidelines for evaluating their viability in carbon capture are a crucial research gap. We address this gap by developing a hierarchical guideline, based on fundamental gas–solid interaction strength, that underpins the success and scalability of AC-based materials. The most critical performance indicator is the CO_2_ adsorption energy, where an optimal range (−0.41 eV) ensures efficiency between adsorption and desorption. Additionally, we consider thermal stability and defect sensitivity to ensure consistent performance under varying conditions. Further, selectivity and capacity play significant roles due to external variables such as partial pressure of CO_2_ and other ambient air gases (N_2_, H_2_O, O_2_), bridging the gap between theory and reality. We provide actionable examples by narrowing our options to methylamine- and pyridine-grafted graphene.

Rapid anthropogenic climate change is one of the central challenges in the 21st century, with atmospheric CO_2_ concentration exceeding 400 ppm.^1−3^ CO_2_ concentration must be maintained at ≤ 450 ppm to mitigate the climate change crisis, which requires capture on the gigaton scale.^4^ This target has necessitated urgent research efforts in the field of carbon capture from both concentrated sources of CO_2_ emissions as well as direct air capture from the atmosphere.^5,6^ Activated carbon (AC)-based materials have shown promising carbon capture performance.^6−8^ They can be inexpensive when sustainably sourced from amply available natural resources such as Balsa wood and Biochar.^9−12^ Moreover, both Balsa wood and Biochar have porous microstructures that can be enhanced multifold and chemically modified during the preparation process to further augment CO_2_ adsorption.^9,13−16^ Overall, their low-cost preparation, hydrophobicity, porosity, and chemical tunability make them attractive solutions for carbon capture via a steam-assisted temperature vacuum swing desorption process.^17^Figure 1 depicts the carbon capture process where wood-derived AC is used as the CO_2_ adsorbent, which is regenerated when subject to high temperature steam. The adsorbed CO_2_ is then separated from water and sent for storage or conversion, while the water is recycled for the next cycle. Furthermore, the adsorption and regeneration system can be strategically coupled with integrated dynamic energy supply systems where excess energy during periods of lower demand can be diverted for sorbent regeneration to provide a more sustainable approach to carbon capture. By designing ACs for optimal CO_2_ adsorption and integrating them with a dynamic energy supply, we can potentially unleash a new generation of relatively cheap, energy-efficient, and highly effective solutions for carbon capture.

**Figure 1:**
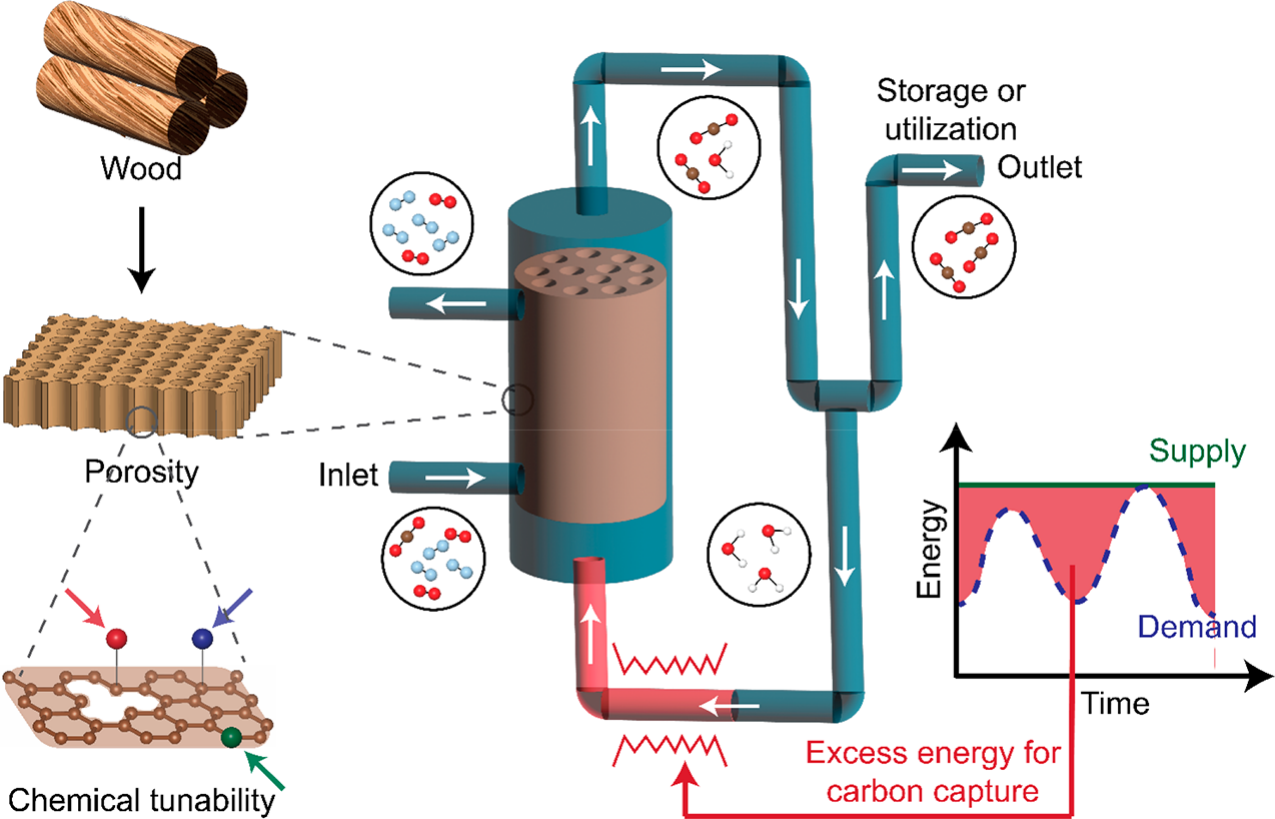
Schematic showing the steam-assisted temperature vacuum swing desorption process with wood-derived AC as the CO_2_ adsorbent. Graphene-derived materials are suitable templates for evaluating activated carbon-based materials. Integrating with a dynamic energy supply results in energy- and cost-effective solutions for carbon capture.

Despite extensive interest in ACs as a new class of materials themselves,^6−8^ a comprehensive theoretical guideline is essential for gauging their viability. Such a theoretical guideline is critically missing. In this work, we made strides in this direction by formulating a list of crucial criteria that underpins the success and scalability of AC-based materials. The single most important parameter determining performance is the CO_2_ adsorption energy (*E_ads_*). Optimum performance necessitates an ideal range of *E_ads_* where CO_2_ is bound neither too strongly for ease of desorption nor too weakly for good selectivity. We used Density Functional Theory (DFT) to evaluate *E_ads_* of CO_2_ on graphene, the ideal surrogate model for present studies^18^ (see Figure 2), with select dopants, functional groups, and defects. To determine the target *E_ads_* accurately, the entire parameter space should be explored by using a consistent computational framework. While there have been multiple DFT studies that calculated CO_2_*E_ads_* on graphene-based materials,^18−22^ none of them have explored the entire parameter space thoroughly, as summarized in Figure S1. To bridge these gaps, we used a consistent computational setup to construct a clear knowledge map of CO_2_ adsorption on graphene-derived materials and opted for a target CO_2_*E_ads_* based on the parasitic energy metric benchmarked with the state-of-art solid-sorber Mg-MOF-74.^23,24^

**Figure 2:**
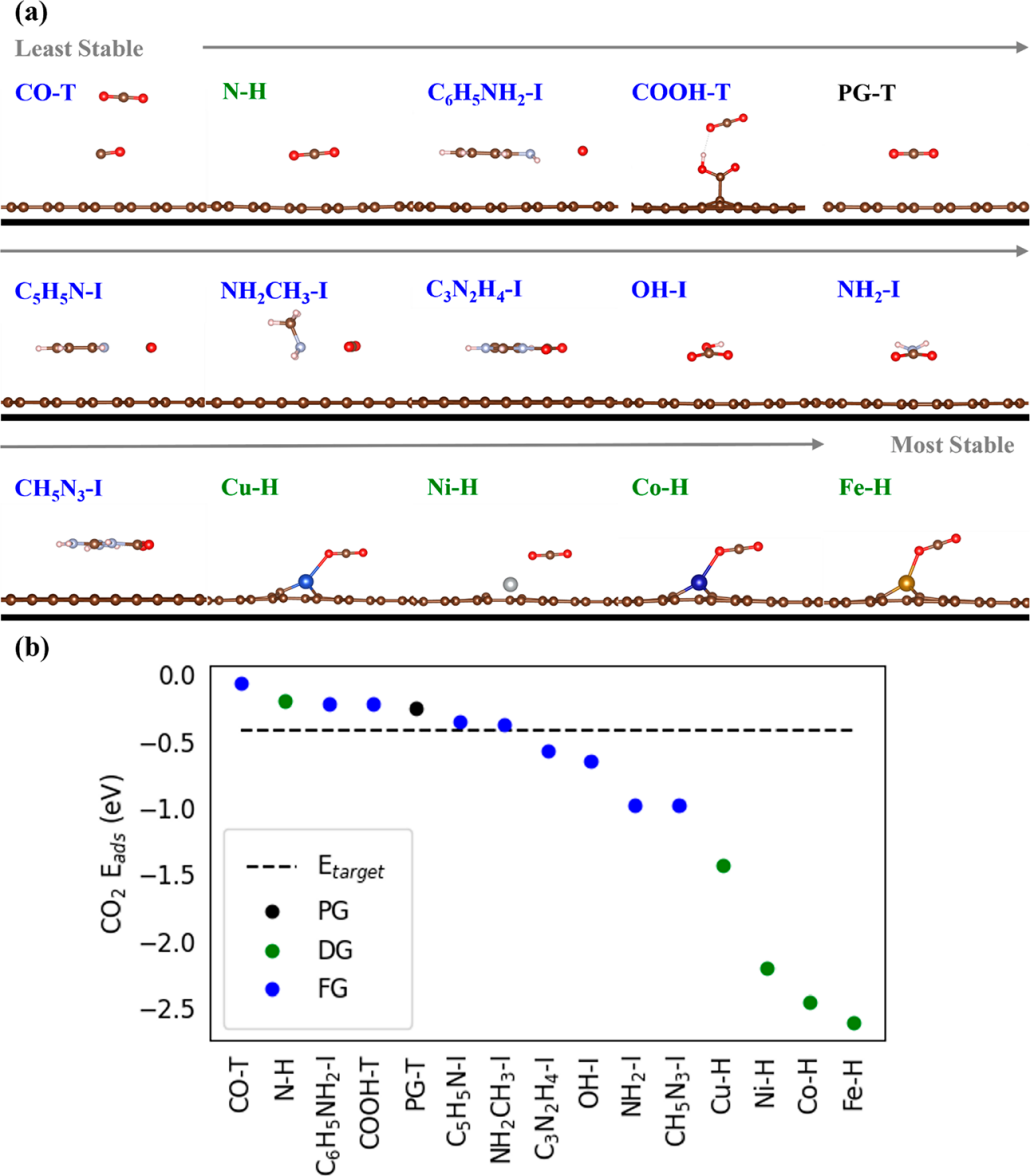
Dopants and functional molecules (FMs) screened in order from least to most stable CO_2_*E_ads_* (a) and their corresponding CO_2_*E_ads_* (b). The most stable CO_2_ configurations on pristine graphene (PG), doped graphene (DG), and functionalized graphene (FG) are either top (T) or hollow (H) sites and T or inserted (I) sites, respectively. *E_ads_* of the most stable sites is reported here, whereas the comprehensive results of all sites can be found in Tables S1 and S3 and Figures S3–S8 and S20–S28.

We then addressed secondary criteria, including thermal stability, defect sensitivity, CO_2_ capacity, and selectivity, to aid in determining the best performing graphene-derived materials for carbon capture and release. Careful considerations mandate that the carbon capture material remain thermally stable and display defect-insensitive performance. This secondary tier of the stability criteria ensures continued performance in the presence of variables such as temperature and defects that are intrinsic to the material. We urge that future studies consider these additional criteria along with adsorption energies to obtain a holistic picture of the viability of the carbon capture material. On the other hand, selectivity and capacity dictate the performance when subjected to variables extrinsic to the material such as partial pressure of CO_2_ and other ambient air gases. These parameters constitute the tertiary tier of requirements that characterize performance in the presence of gases simulating a realistically complex carbon capture system. Finally, other considerations like synthesizability, economic feasibility, and life cycle analysis serve as the last check before a technology can be scaled up successfully.

CO_2_*E_ads_* is the primary metric to determine the best performing graphene-derived materials due to its inherent nature of containing the energy required to release CO_2_ (see eq 1)
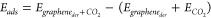
1where *E*_*graphene*_*der*_ + *CO*_2__, *E*_*graphene*_*der*__, and *E*_*CO*_2__ are the total energies of their relaxed structures. The chemisorption threshold is commonly accepted between −0.41 and −0.51 eV,^25^ where more stable (more negative) is chemisorption and less stable (less negative) is physisorption. The less stable boundary of this threshold range is consistent with our target CO_2_*E_ads_*, which is motivated by the parasitic energy metric,^23^ based on monoethanolamine, an industry standard,^24^ used to screen covalent organic frameworks (COFs) for carbon capture in Deeg et al.^24^ Parasitic energy contains the energy required to separate CO_2_ from flue gas (*Q*_*separation*_), which should be minimized to reduce energy losses resulting in a CO_2_ heat of adsorption threshold of −0.46 eV.^24^ Further, the best COFs are comparable to Mg-MOF-74 (metal organic framework). A DFT calculation of CO_2_*E_ads_* on Mg-MOF-74 is −0.41 eV (see Figure S2), which is assigned as our target CO_2_*E_ads_*.

To determine materials that satisfy our primary criteria, a wide selection guided by electronegativity (EN) difference was chosen, and CO_2_*E_ads_* was evaluated as indicated in Figure 2 (see Tables S1 and S3 and Figures S3–S8 and S20–S28 for all CO_2_ sites and *E_ads_*). First, the baseline case of CO_2_ adsorption on pristine graphene (PG) was evaluated and found to be less stable than that of our target. Next, N was substitutionally doped due to its (∼0.5 )^26^ EN difference with C, but it resulted in weak physisorption. In addition, this result ruled out B and P as dopants due to their smaller EN difference with C in graphene-based materials than dopants such as O and N. Therefore, we expect O and N to be better representatives of dopants for this system. Thereafter, we increased the EN difference with O (>1.0 )^26^ by moving to transition metal doping, but this showed too strong chemisorption. Next, for a lower EN difference (>0.9 )^26^ with CO_2_, we moved to O-containing functional molecules (FMs) adsorbing in the basal plane on PG. CO_2_ physisorbed on CO and COOH functionalized graphene (FG), while CO_2_ chemisorbed on OH FG and formed bicarbonate. Overall, graphene derivatives with dopants and O-containing FMs drove the CO_2_*E_ads_* farther from our target.

To get closer to our target CO_2_*E_ads_*, we reduced the EN difference with CO_2_ by moving to N-containing FMs. CO_2_ physisorbed with aniline FG, while CO_2_ chemisorbed with amine, guanidine, and imidazole FG. All of these are sufficiently far from our target, but aniline and amine provide a platform for further improvement. In the amine case, we decreased the EN difference with CO_2_ by moving to methylamine FG, which adsorbed CO_2_ near our target. Similarly, we moved from aniline to pyridine, in which the latter met the CO_2_*E_ads_* target. To understand further, we investigated the Bader charge difference (BCD) and charge density difference (CDD) for a few N-containing FMs (Figure 3 and Tables S10–S15). Interestingly, CO_2_ is more stable interacting with amine in the inserted site over the top site as the former shows a higher amount of BCD compared with the latter. The other N-containing FMs also prefer to interact with CO_2_ in the inserted sites. Significant BCD is seen in amine and guanidine due to bonding but not in pyridine and methylamine which fall in the target *E_ads_* range, which further shows the unique positions of pyridine and methylamine at the boundary between physisorption and chemisorption as desired.

**Figure 3:**
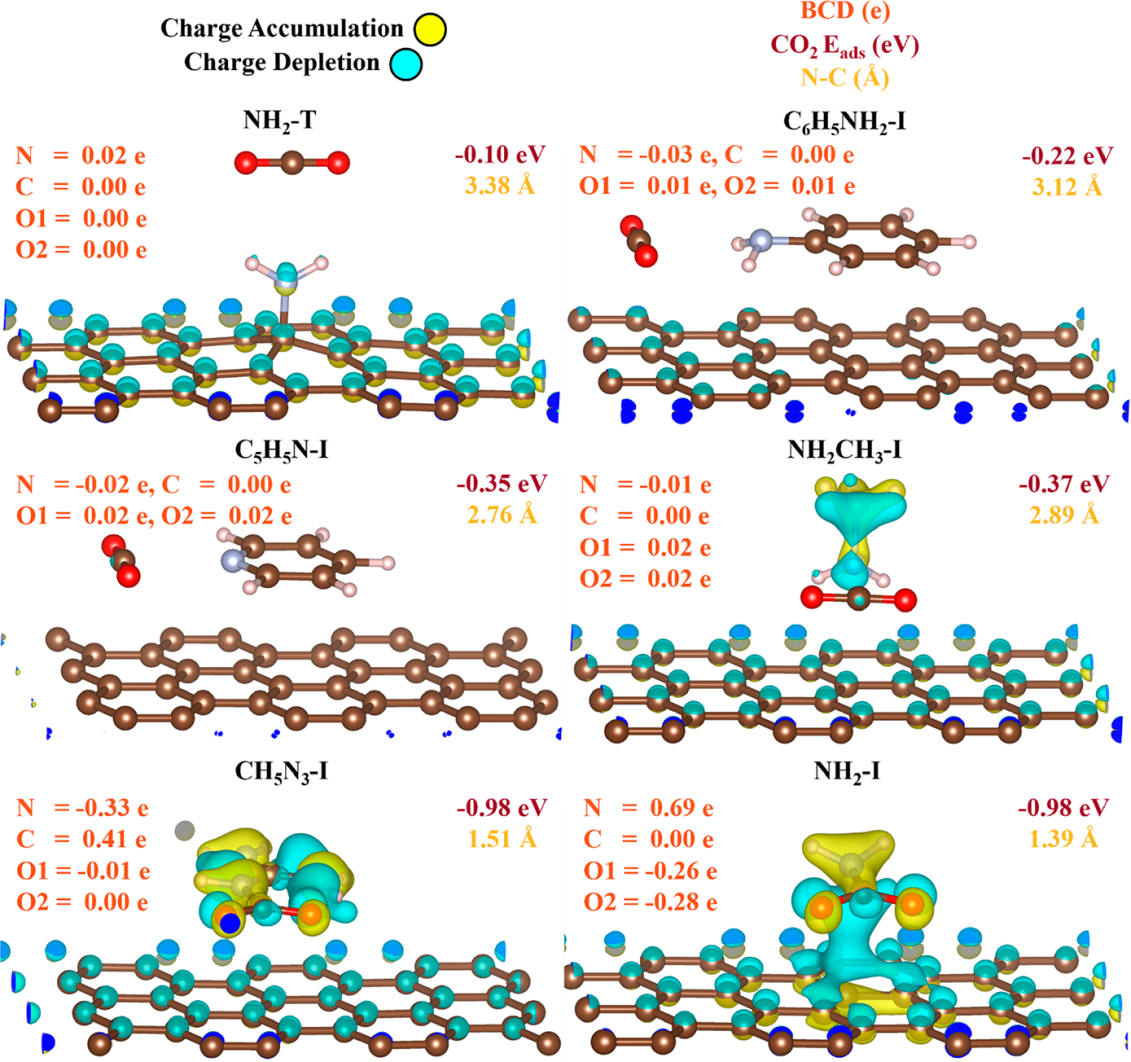
Charge density difference (CDD) plots, Bader charge difference (BCD), CO_2_*E_ads_*, and distances between N (of functional molecule (FM)) and C (of CO_2_) for graphene-derived materials (GDM) in top (T) and inserted (I) sites. BCD is reported for N (of FM), C (of CO_2_), and both O’s (of CO_2_) with the top/left as the O1 atom and the right/bottom as the O2 atom.

While the primary criteria of target CO_2_*E_ads_* ensure an ideal adsorption condition, other guidelines should also be considered to enable the viability of this technology. The secondary tier of criteria governs the intrinsic properties of the material that is essential for performance. It accounts for ineradicable material defects and ensures thermal stability. We define thermal stability as the ability of a material to stay unaltered during the CO_2_ desorption process. For thermal stability, the FM must bind with PG in the chemisorption range to eliminate its removal post CO_2_ desorption. While a general guideline is hard to propose, a higher *E_ads_* (in the chemisorption range) of the FM on PG compared to the CO_2_*E_ads_* on the corresponding FG is needed to ensure thermal stability. Thus, we calculated the *E_ads_* of FMs on PG (see Figure 4b, Table S2, and Figures S9–S19). Pyridine adsorbed onto PG in the chemisorption range and is, therefore, thermally stable. However, methylamine is less stable on PG than CO_2_, revealing it will desorb with CO_2_ and provide an impure CO_2_ stream. An impure CO_2_ stream may be acceptable if the stakeholders use this technology for sequestration. Moreover, manufacturing materials completely free of defects is extremely difficult. We define a defect insensitive material as a desirable material whose CO_2_*E_ads_* does not change when defects are introduced. In other words, the CO_2_*E_ads_* values of the pristine material and the material with defects are the same. Thus, to ensure that the properties of the capture material are unaffected by the presence of defects, CO_2_*E_ads_* must be invariant between PG and defect graphene. We considered monovacancy graphene (MG) defects as they alter CO_2_*E_ads_* more than any other graphene defect (like the Stone–Wales defect)^19,27^ due to the dangling C bonds interacting more strongly with CO_2_ (see Figure 4c, Table S5, and Figure S31). Methylamine adsorbs onto MG near the lower chemisorption threshold (see Table S4 and Figure S29) while adsorbing CO_2_ near our target CO_2_*E_ads_* (see Figure 4c, Table S5, and Figure S32) and is therefore a defect insensitive material. However, pyridine forms two C–C bonds with MG and is much more stable than on PG (see Table S4 and Figure S30), but pyridine is near perpendicular to MG resulting in a less stable CO_2_*E_ads_* when compared to pyridine FG (see Figure 4c, Table S5, and Figure S33). Overall, while pyridine and methylamine satisfy the primary criterion of being near the target CO_2_*E_ads_*, they fail the material defects and thermal stability criteria, respectively, indicating the importance of considering such secondary factors in the evaluation criteria.

**Figure 4:**
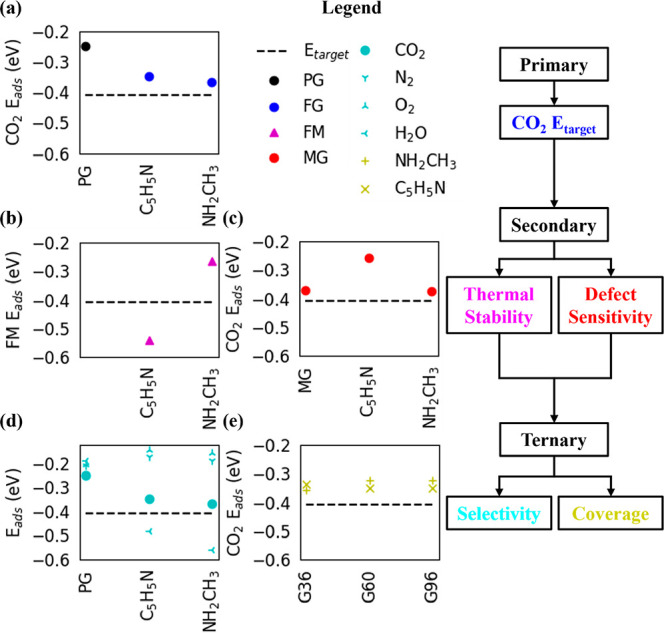
CO_2_*E_ads_* (a) of most stable CO_2_ configurations on pristine graphene (PG) and functionalized graphene (FG), (b) thermal stability by functional molecule (FM) *E_ads_* on PG, (c) defect sensitivity by CO_2_*E_ads_* on monovacancy graphene (MG), (d) selectivity by CO_2_, N_2_, O_2_, and H_2_O *E_ads_*, and (e) coverage effects by CO_2_*E_ads_* (single point calculations without ionically relaxing PG) on G36 (36 C atoms), G60 (60 C atoms), and G96 (96 C atoms) PG sheets.

Lastly, ternary selection criteria are important to consider like selectivity and capacity, which control the performance of the material due to extrinsic parameters like the presence of ambient air gases. While selectivity will be dependent on the sticking coefficient, kinetic diameter, and pore size of the material in the realistic carbon capture setting, the comparative CO_2_*E_ads_* is a necessary parameter to investigate at the DFT level (see Table S6 and Figures S34–S42). PG energetically favors CO_2_ over H_2_O, O_2_, and N_2_ (see Figure 4d). On methylamine and pyridine FG, O_2_ and N_2_ prefer to adsorb onto PG, leaving the inserted sites near the FM open for CO_2_. However, H_2_O is more stable than CO_2_ on methylamine and pyridine FG due to N–H bonding in the inserted sites, which opens the door for either cooperative or competitive adsorption (see Table S7 and Figures S43–S45). When CO_2_ and H_2_O were both allowed to adsorb, adsorbates such as bicarbonate and carbonic acid can also form. For methylamine FG, CO_2_ forms carbonic acid with H_2_O, which then dissociates H to NH_2_CH_3_ and forms NH_3_CH_3_^+^ and bicarbonate. Further, bicarbonate adsorption on NH_3_CH_3_^+^ FG is much more stable than CO_2_ adsorption on H_2_O adsorbed methylamine FG, followed with bicarbonate and NH_3_CH_3_^+^ adsorption on PG. Thus, bicarbonate and NH_3_CH_3_^+^ will desorb from PG together as they are the least stable of the three interactions. For pyridine FG, CO_2_ and H_2_O form carbonic acid and interact strongly with pyridine due to the N–H bond. Similar to methylamine FG, pyridine and carbonic acid will desorb together, as their *E_ads_* on PG is less than that of carbonic acid on pyridine. Although both methylamine and pyridine FG support cooperative adsorption with H_2_O and CO_2_, both FMs desorb with either bicarbonate or carbonic acid, leading to an impure CO_2_ stream, and therefore fail the thermal stability requirement in the secondary criteria.

The variation of CO_2_*E_ads_* with increasing adsorption sites is an important factor for the CO_2_ capacity. Here the number of available adsorption sites was changed by varying the number of FMs in a given PG area. We used the Langmuir Isotherm^28,29^ model to predict CO_2_ coverage at select CO_2_ concentrations (see equation S1). To study the baseline case of PG, we calculated CO_2_*E_ads_* on PG in sheets of 36, 252, 780, and 1152 C atoms (see Table S8 and Figure S46). CO_2_*E_ads_* is well-converged for all PG sheet sizes when only considering enthalpy. In addition, we calculated CO_2_*E_ads_* on methylamine and pyridine FG at select coverages (see Figure 4e, Table S8, and Figures S47 and S48). van der Waals interactions are minimal at 7 Å and negligible > 10 Å, and these distances correspond to 60 and 96 C atom sheets, respectively, for both methylamine and pyridine FG. CO_2_*E_ads_* is converged with both methylamine and pyridine FG at a coverage of 60 C atoms/CO_2_ molecules (15000 ppm). Interestingly, CO_2_ is more stable on the 36 C atom sheet (than 60 and 96) of methylamine FG due to an attractive O–H interaction between CO_2_ and methylamine in repeating images. On the contrary, CO_2_ is less stable on the 36 C atom sheet (than 60 and 96) of pyridine FG due to repulsive H–H interactions of pyridine in repeating images. Enhanced attractive interactions are seen in higher methylamine and lower pyridine coverages, indicating preferred CO_2_ adsorption at those coverages. This highlights the importance of evaluating FG coverage in *E_ads_* studies as a precursor to understanding capacity.

Traditionally, only *E_ads_* has been utilized as the sole parameter for evaluating carbon capture performance,^24^ but this work defines the target value and shows how comprehensive perspectives like thermal stability, defect sensitivity, selectivity, and capacity can have a defining role in ensuring functionality. While methylamine and pyridine pass the primary criteria, they fail at some of the secondary and ternary ones. Moreover, ternary parameters like selectivity underscore the complex interactions that are often at play which can decisively impact the performance as demonstrated by the creation of adsorbates like bicarbonate following cooperative adsorption. These adsorbates can have an adverse effect on other factors such as thermal stability as highlighted by the instability of both pyridine and methylamine upon carbonic acid and bicarbonate formation. Merely considering only the primary criteria overlooks the complexity of the chemical space and can lead to erroneous predictions. Thus, we highly recommend considering these secondary and ternary factors when making predictions for carbon capture in the future.

In summary, we designed a comprehensive theoretical guideline consisting of a hierarchy of crucial criteria that maps the complex carbon capture challenge to a set of fundamental physical chemistry properties. The primary criteria of optimal CO_2_*E_ads_* is followed by secondary ones like thermal stability and material defects and succeeded by ternary parameters like selectivity and capacity. To ensure optimum performance, we opted for a target CO_2_*E_ads_* of −0.41 eV that represents the desired boundary between physisorption and chemisorption and aligns well with state-of-art Mg-MOF74 CO_2_ adsorption strength (see more in the SI). Moreover, a good candidate material must also be thermally stable during the CO_2_ desorption process. Only those functional groups which adsorbed on graphene with a higher energy (<−0.41 eV) compared to the adsorption energy of CO_2_ on graphene functionalized with that group passed this criterion of thermal stability. Since defects are unavoidable, it is imperative that the CO_2_*E_ads_* remains unchanged in the presence of defects. Additionally, a good candidate material must also preferentially adsorb CO_2_ over other gases. In other words, the selectivity criterion requires the CO_2_*E_ads_* to be higher than the rest. Finally, the CO_2_*E_ads_* can also vary with the density or coverage of the functional groups, and for optimum capacity, the coverage resulting in the highest CO_2_*E_ads_* should be chosen.

Based on the devised criteria and to provide practical guidance, we computed the *E_ads_* of a variety of FMs and dopants on PG with the objective of exploring the *E_ads_* parameter space and designing suitable AC-based adsorbents for CO_2_ capture and release. By using a consistent computational setup, we ensured that all the results are reproducible and comparable. We found that methylamine and pyridine adsorb CO_2_ near the target. We showed that since defects in the structure are unavoidable, for optimal performance, the CO_2_*E_ads_* should be relatively insensitive to these defects. Additionally, the FMs should be more strongly bound than CO_2_ to PG to ensure thermal stability during CO_2_ desorption. While cooperative adsorption between CO_2_ and H_2_O on methylamine and pyridine FG is predicted, it is accompanied by thermal instability. Lastly, the variation of CO_2_*E_ads_* with FG coverage translated to a lower pyridine and higher methylamine coverage for enhanced adsorption. This work sheds light on the necessity to evaluate FMs on secondary and ternary requirements. We propose that our approach creates a pathway to evaluate materials for carbon capture systems and that consideration of FG-like surfaces are a promising platform. Future work on carbon capture using adsorbent technology should also consider these additional requirements. Furthermore, this theoretical guideline can be generalized to different carbon capture materials, like other 2D materials (MoS_2_, WSe_2_, CrO_2_, CrS_2_, VO_2_, VS_2_, h-BN, NbSe_2_, etc.), since the list of criteria devised for CO_2_ capture that include optimum *E_ads_*, thermal stability, defect insensitivity, high selectivity, and capacity is material agnostic. CO_2_*E_ads_*, the primary parameter that all the other criteria are dependent on, in turn relies on the knowledge of fundamental interactions between CO_2_ and the solid-sorber of interest. Such studies will play a critical role in the future designs of the CO_2_ capture solutions.

## Supporting Information Available

Additional calculation details in figures and tables along with computational methods (PDF)
Transparent Peer Review report available (PDF)
