# Supplementary material for: Carbon Capture: Theoretical Guidelines for Activated Carbon-Based CO2 Adsorption Material Evaluation
Source: J Phys Chem Lett. 2023 Nov 21;14(47):10693–9. doi: 10.1021/acs.jpclett.3c02711 (PMC10694831; doi:10.1021/acs.jpclett.3c02711)
Supplement: Supplementary file 1 — jz3c02711_si_001.pdf [file jz3c02711_si_001.pdf]

Supplementary Information:

Carbon Capture: Theoretical Guidelines for Activated Carbon-based CO_2 Adsorption Material Evaluation

Drew M. Glenna#[a], Asmita Jana#[b, c], Qiang Xu[b], Yixiao Wang[d, e], Yuqing Meng[d], Yingchao Yang[f, g], Manish Neupane[f, g], Lucun Wang[d], Haiyan Zhao*[a, h], Jin Qian*[b], and Seth W. Snyder*[d]

[a] Department of Nuclear Engineering & Industrial Management, University of Idaho, Idaho Falls, ID 83402, USA

[b] Chemical Sciences Division, Lawrence Berkeley National Laboratory, Berkeley, CA 94720, USA

[c] Advanced Light Source, Lawrence Berkeley National Laboratory, Berkeley, CA 94720, USA

[d] Energy & Environmental Science and Technology, Idaho National Laboratory, Idaho Falls, ID 83415, USA

[e] Current Address: School of Physical Science and Technology, ShanghaiTech University, Shanghai, 201210, China

[f] Department of Mechanical Engineering, University of Maine, Orono, ME 04469, USA

[g] Current Address: Mechanical & Aerospace Engineering, College of Engineering, University of Missouri, Columbia, MO 65201, USA

[h] Department of Chemical and Biological Engineering, University of Idaho, Idaho Falls, ID 83402, USA
E-mail: haiyanz@uidaho.edu, jqian2@lbl.gov, seth.snyder@inl.gov

Table of Contents
Computational Methods....................5
Geometry Set Up....................5
DFT Calculation Method....................5
Charge Analysis Method....................5
Physisorption-Chemisorption Boundary on Graphene Materials....................6
Summary of the state-of-the-art DFT calculation results on graphene derivatives....................6
Figure S1. Current state-of-the-art DFT calculations of CO_2 E_{ads} on PG, DG, FG, MG, and Stone-Wales defect (SWG) graphene....................7

CO_2 E_{ads} on Mg-MOF-74 7
Figure S2. CO_2 E_{ads} on Mg-MOF-74 8
CO_2 Adsorption on Doped Graphene (DG) 8
Table S1. CO_2 E_{ads} on PG and DG with shortest atomic distances 8
Figure S3. Ionically relaxed CO_2 configuration on PGT (a, b) and PGH (c, d) sites 9
Figure S4. Ionically relaxed CO_2 configuration on NGT1 (a, b), NGT2 (c, d), NGT3 (e, f), NGTP (g, h), and NGH (i, j) sites 10
Figure S5. Ionically relaxed CO_2 configuration on FeGT (a, b), FeGTP (c, d), and FeGH (e, f) sites 10
Figure S6. Ionically relaxed CO_2 configuration on CoGT (a, b) and CoGH (c, d) sites 11
Figure S7. Ionically relaxed CO_2 configuration on NiGT1 (a, b), NiGT2 (c, d), NiGH1 (e, f), and NiGH2 (g, h) sites 11
Figure S8. Ionically relaxed CO_2 configuration on CuGT1 (a, b), CuGT2 (c, d), CuGH1 (e, f), CuGH2 (g, h), and CuGH3 (i, j) sites 12
CO_2 Adsorption on Functionalized Graphene (FG) 12
Table S2. FMs E_{ads} on PG with shortest atomic distances 14
Table S3. CO_2 E_{ads} on FG with shortest atomic distances between FM and CO_2 (elements are in parentheses if a bond is formed), CO_2 and PG (y (vacuum) direction), and FM and PG 15
Figure S9. Ionically relaxed CO on PG 16
Figure S10. Ionically relaxed OH on PG 16
Figure S11. Ionically relaxed COOH on PG 16
Figure S12. Ionically relaxed NH_2 configuration on PG in NH_2T (a, b), NH_2H (c, d), and NH_2B (e, f) sites 17
Figure S13. Ionically relaxed NH_2CH_3 configuration on PG in NH_2CH_3T1 (a, b), NH_2CH_3T2 (c, d), NH_2CH_3T3 (e, f), NH_2CH_3T4 (g, h), NH_2CH_3H1 (i, j), NH_2CH_3H2 (k, l), NH_2CH_3H3 (m, n), NH_2CH_3B1 (o, p), and NH_2CH_3B2 (q, r) sites 17
Figure S14. Ionically relaxed $NHCH_3$ configuration on PG in $NHCH_3$T1 (a, b) and $NHCH_3$T2 (c, d) sites 18
Figure S15. Ionically relaxed NH_2CH_2 configuration on PG in NH_2CH_2T (a, b) site 18
Figure S16. Ionically relaxed C_5H_5N configuration on PG in C_5H_5NT (a, b), C_5H_5NH (c, d), and C_5H_5NB (e, f) sites 18
Figure S17. Ionically relaxed $C_6H_5NH_2$ configuration on PG in $C_6H_5NH_2$H1 (a, b), $C_6H_5NH_2$H2 (c, d), and $C_6H_5NH_2$B (e, f) sites 19
Figure S18. Ionically relaxed CH_5N_3 configuration on PG in CH_5N_3TT (a, b), CH_5N_3TH (c, d), CH_5N_3HT (e, f), and CH_5N_3HB (g, h) sites 19

Figure S19. Ionically relaxed $C_3N_2H_4$ configuration on PG in $C_3N_2H_4$T1 (a, b), $C_3N_2H_4$T2 (c, d), $C_3N_2H_4$TT1 (e, f), $C_3N_2H_4$TT2 (g, h), $C_3N_2H_4$TT3 (i, j), and $C_3N_2H_4$H (k, l) sites 20

Figure S20. Ionically relaxed CO_2 configuration on COG 20

Figure S21. Ionically relaxed CO_2 configuration on OHGT (a, b) and OHGi (c, d) sites 20

Figure S22. Ionically relaxed CO_2 configuration on COOHG1 (a, b (xy plane)) and COOHG2 (c, d) sites 21

Figure S23. Ionically relaxed CO_2 configuration on NH_2GT1 (a, b), NH_2GT2 (c, d), NH_2Gi1 (e, f), and NH_2Gi2 (g, h) sites 21

Figure S24. Ionically relaxed CO_2 configuration on NH_2CH_3GT (a, b), NH_2CH_3Gi1 (c, d), NH_2CH_3Gi2 (e, f), and NH_2CH_3Gi3 (g, h) sites 22

Figure S25. Ionically relaxed CO_2 configuration on C_5H_5NGT (a, b), C_5H_5NGi1 (c, d), and C_5H_5NGi2 (e, f) sites............22

Figure S26. Ionically relaxed CO_2 configuration on $C_6H_5NH_2$GT1 (a, b), $C_6H_5NH_2$GT2 (c, d), $C_6H_5NH_2$Gi1 (e, f), $C_6H_5NH_2$Gi2 (g, h), and $C_6H_5NH_2$Gi3 (i, j) sites 23

Figure S27. Ionically relaxed CO_2 configuration on CH_5N_3GT (a, b), CH_5N_3Gi1 (c, d), CH_5N_3Gi2 (e, f), and CH_5N_3Gi3 (g, h) sites 24

Figure S28. Ionically relaxed CO_2 configuration on $C_3N_2H_4$GT (a, b), $C_3N_2H_4$Gi1 (c, d), and $C_3N_2H_4$Gi2 (e, f) sites 24

CO_2 Adsorption on Monovacancy Defect Graphene (MG) 24

Table S4. FMs E_{ads} on MG with shortest atomic distances 25

Table S5. CO_2 E_{ads} on FG on MG with shortest atomic distances between FM and CO_2 (elements are in parentheses if a bond is formed), CO_2 and PG (y (vacuum) direction), and FM and PG25

Figure S29. Ionically relaxed NH_2CH_3 configuration on MG in NH_2CH_3MG1 (a, b) and NH_2CH_3MG2 (c, d) sites............26

Figure S30. Ionically relaxed C_5H_5N configuration on MG in C_5H_5NMGT1 (a, b), C_5H_5NMGT2 (c, d), C_5H_5NMGH (e, f), C_5H_5NMGB1 (g, h), C_5H_5NMGB2 (i, j), and C_5H_5NMGB3 (k, l) sites 26

Figure S31. Ionically relaxed CO_2 configuration on MG1 (a, b), MG2 (c, d), MG3 (e, f), and MG4 (g, h) sites 27

Figure S32. Ionically relaxed CO_2 configuration on MG1NH_2CH_3 (a, b) and MG2NH_2CH_3 (c, d) sites 27

Figure S33. Ionically relaxed CO_2 configuration on MGC_5H_5NT1 (a, b), MGC_5H_5NT2* (c, d), MGC_5H_5Ni1 (e, f), MGC_5H_5Ni2 (g, h), and MGC_5H_5Ni3 (i, j) sites 28

Selectivity with E_{ads} 28

Table S6. N_2, O_2, and H_2O E_{ads} on FG with shortest atomic distances between FM and gas (elements are in parentheses if a bond is formed), gas and PG (y (vacuum) direction), and FM and PG............28

Figure S34. Ionically relaxed H_2O configuration on PG in H_2OGT (a, b), H_2OGH (c, d), and H_2OGB (e, f) sites 30

Figure S35. Ionically relaxed H_2O configuration on GNH_2CH_3 in $H_2OGNH_2CH_3T$ (a, b), $H_2OGNH_2CH_3i1$ (c, d), and $H_2OGNH_2CH_3i2$ (e, f) sites 30

Figure S36. Ionically relaxed H_2O configuration on GC_5H_5N in $H_2OGC_5H_5NT1$ (a, b), $H_2OGC_5H_5NT1$ (c, d), $H_2OGC_5H_5Ni1$ (e, f), and $H_2OGC_5H_5Ni2$ (g, h) sites 31

Figure S37. Ionically relaxed O_2 configuration on PG in O_2GT (a, b), O_2GH (c, d), and O_2GB (e, f) sites 31

Figure S38. Ionically relaxed O_2 configuration on GNH_2CH_3 in $O_2GNH_2CH_3T$ (a, b), $O_2GNH_2CH_3i1$ (c, d), and $O_2GNH_2CH_3i2$ (e, f) sites 32

Figure S39. Ionically relaxed O_2 configuration on GC_5H_5N in $O_2GC_5H_5NT1$ (a, b), $O_2GC_5H_5NT2$* (c, d), $O_2GC_5H_5Ni1$* (e, f), and $O_2GC_5H_5Ni2$ (g, h) sites 33

Figure S40. Ionically relaxed N_2 configuration on PG in N_2GT1 (a, b), N_2GT2 (c, d), N_2GH1 (e, f), N_2GH2 (g, h), and N_2GB (i, j) sites 33

Figure S41. Ionically relaxed N_2 configuration on GNH_2CH_3 in $N_2GNH_2CH_3T$ (a, b), $N_2GNH_2CH_3i1$ (c, d), and $N_2GNH_2CH_3i2$ (e, f) sites 33

Figure S42. Ionically relaxed N_2 configuration on GC_5H_5N in $N_2GC_5H_5NT1$ (a, b), $N_2GC_5H_5NT1$ (c, d), $N_2GC_5H_5Ni1$* (e, f), and $N_2GC_5H_5Ni2$ (g, h) sites 34

Coadsorption with E_{ads} 34

Table S7. CO_2 E_{ads} on H_2O adsorbed FG with shortest atomic distances between FM and gas (elements are in parentheses if a bond is formed), gas and PG (y (vacuum) direction), and FM and PG 35

Figure S43. Ionically relaxed CO_2 configuration on $H_2OGNH_2CH_3$ in $CO_2{:}H_2OGNH_2CH_3i$ (a, b) site 35

Figure S44. Ionically relaxed CO_2 configuration on $H_2OGC_5H_5N$ in $CO_2{:}H_2OGC_5H_5NT$ (a, b), $CO_2{:}H_2OGC_5H_5Ni1$ (c, d), $CO_2{:}H_2OGC_5H_5Ni2$ (e, f), $CO_2{:}H_2OGC_5H_5Ni3$ (g, h), and $CO_2{:}H_2OGC_5H_5Ni4$ (i, j) sites 36

Figure S45. Ionically relaxed H_2CO_3 configuration on GC_5H_5N in $H_2CO_3GC_5H_5Ni$ (a, b) site 36

Coverage Effects on CO_2 E_{ads} 36

Table S8. CO_2 E_{ads} (eV) on PG, NH_2CH_3-FG, and C_5H_5N-FG with respect to coverage 37

Figure S46. Single point configuration of CO_2 on PG in 36 (a), 252 (b), 780 (c), and 1152 (d) C atom sheets using ARES 38

Figure S47. Single point configuration of CO_2 on GNH_2CH_3 in 36 (a), 60 (b), and 96 (c) C atom sheets using QE 38

Figure S48. Single point configuration of CO_2 on GC_5H_5N in 36 (a), 60 (b), and 96 (c) C atom sheets using QE 39

Van der Waals and Electrostatic Contributions in CO_2 E_{ads} 39
Table S9. Interaction energies (eV) of CO_2 with FMs and PG. 39
Bader Charge Analysis 40
Table S10. Bader Charge Difference (BCD (e)) for NH_2-T FG 40
Table S11. BCD (e) for $C_6H_5NH_2$-I FG 41
Table S12. BCD (e) for C_5H_5N-I FG 42
Table S13. BCD (e) for NH_2CH_3-I FG 44
Table S14. BCD (e) for CH_5N_3-I FG 45
Table S15. BCD (e) for NH_2-I FG 46

Computational Methods

Geometry Set Up

A single layer of pristine graphene (PG) is modeled using the Atomic Simulation Environment[1] (ASE) as a 3 × 3 × 1 supercell with 36 C atoms. The PG layer is modeled as a sheet with x and z being the periodic directions. To avoid interactions between PG sheets a vacuum gap of 15 Å is employed in the y axis. The lattice parameter is set to 2.452 Å determined by an ionic and volume relaxation calculation. For consistency, CO_2, N_2, O_2, H_2O, and all the functional molecules (FMs) are modeled in the same cell dimensions (7.36 × 12.90 × 15.00 Å^3) as the 36 C atom sheet. Six different sizes (36, 60, 96, 252, 780, and 1152) of C atom sheets were generated with ASE's set PG structure (1.42 Å C-C distance) to study coverage effects. Each of the 252, 780, and 1152 C atom sheets contain a 5 Å vacuum gap in the x axis, with the dangling edge C atoms saturated with H atoms, to minimize computational cost. The atomic positions of the ionically relaxed methylamine and pyridine FMs (along with CO_2) on PG were used in the 36, 60 and 96 C atom sheet coverage calculations.

DFT Calculation Method

Spin Polarized DFT calculations are performed in Quantum Espresso[2–4] (QE) with van der Waals forces (Grimmes D2)[5,6] to account for long range London dispersion interactions between the adsorbent and adsorbates. Plane-wave basis sets for the valence electron eigenfunctions are used with the projector-augmented[7] method along with the generalized gradient approximation exchange functional by Perdew-Burke-Enzerhof.[8] Gaussian smearing is employed with a smearing width of 0.001 Ry. A Monkhorst-Pack k-point grid of 2 × 1 × 1 with a kinetic energy cutoff of 100 Ry was used for the ionic and volume relaxation calculations with pristine and doped graphene. In these calculations, the x and z directions of the graphene sheet are relaxed to a pressure of 0 bar with a convergence threshold of 0.5 bar. The total energy and force convergence threshold for ionic minimization is 2.72×10^{-3} eV and 2.72×10^{-2} eV, respectively. The total energy convergence threshold for self-consistency is 2.72×10^{-5} eV. A k-point grid of 4 × 2 × 2 and a kinetic energy cutoff of 85 Ry achieved <1 meV/atom convergence for all structures and molecules. The latter grid and cutoff are implemented in all the ionic relaxation and self-consistent field calculations for

the adsorption energy (E_{ads}) calculations in this work. For coverage effects, single point calculations with a gamma point and an 85 Ry cutoff was used.

Charge Analysis Method

Three methods are used to further indicate and describe electron interactions and the type of bonding. First, the interaction distances between CO_2 and the graphene derivative is analyzed to determine if the dominant forces are either van der Waals, Coulombic, or chemical bonding. Second, Bader charge[9–12] is used to determine the depletion and augmentation of charge on the atoms. Third, the charge density difference of CO_2 and the graphene derivative is plotted using VESTA[13] to provide further insight into the depletion and augmentation of charge by visualizing their charge density before and after CO_2 is introduced.

Physisorption-Chemisorption Boundary on Graphene Materials

From a physical chemistry perspective, physisorption typically corresponds to non-bonding interactions like van der Waals whereas chemisorption often involves a significant charge transfer accompanied with formation of chemical bonds. More practically, the boundary between physisorption and chemisorption is commonly accepted to be in the range of -0.41 to -0.51 eV,[14] and we found it to be apt for our system. For instance, the CO_2 adsorption energy of methylamine functionalized graphene is right below the physisorption-chemisorption boundary (-0.367 eV), and we found that there was no N-C bond formed with a distance of 2.893 Å (see Table S3). In addition, CO_2 exhibited no Bader charge transfer with methylamine (see Table S13). On the other hand, imidazole functionalized graphene adsorbed CO_2 with an energy just above the physisorption-chemisorption boundary (-0.574 eV) and formed a N-C bond with a distance of 1.611 Å (see Table S3). Furthermore, CO_2 and imidazole showed Bader charge transfer with N (of $C_3N_2H_4$), C (of CO_2), O1 (of CO_2), and O2 (of CO_2) gaining -0.45, 0.21, 0.11, and 0.07 e, respectively. A similar CO_2 adsorption energy range (-0.41 to -0.78 eV) is commonly accepted for direct air capture applications.[15,16]

Summary of the state-of-the-art DFT calculation results on graphene derivatives

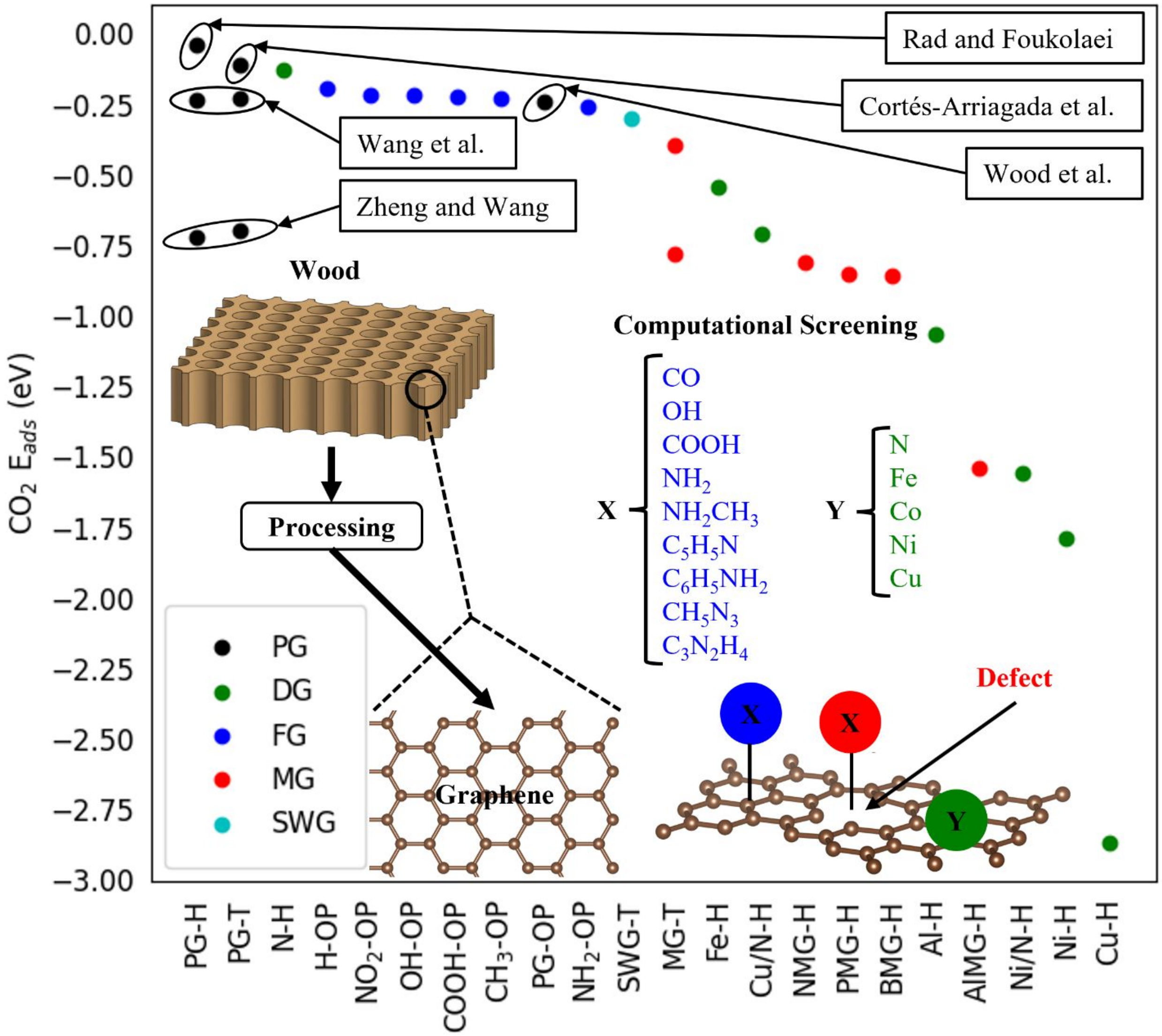

Figure S1. Current state-of-the-art DFT calculations of CO_2 E_{ads} on PG, DG, FG, MG, and Stone-Wales defect (SWG) graphene[17-21] with the process schematic of wood to graphene on the nanoscale including our evaluation of DG, FG, and MG.

CO_2 E_{ads} on Mg-MOF-74

To base off of the ideal CO_2 E_{ads} that is neither too large nor too small, we turned to calculation of the CO_2 adsorption on the current state-of-art solid sober solution: Mg-MOF-74.[22-27] MOF-74 or M_2(dobdc) (H_4dobdc = 2,5-dihydroxyterephthalic acid; M = Mg, Co, Ni, Zn, Mn, Fe) is a benchmark framework with outstanding adsorption properties for a range of applications, including carbon capture. The structure of MOF-74 is generally described as hexagonal honeycomb channels with accessible open metal sites. CO_2 is absorbed through the interaction of O and the Mg site. The CO_2 E_{ads} at the same PBE-D2 level of calculation is -0.41 eV, in good agreement with the results from Vlaisavljevich et.al and Lee et.al.[28,29]

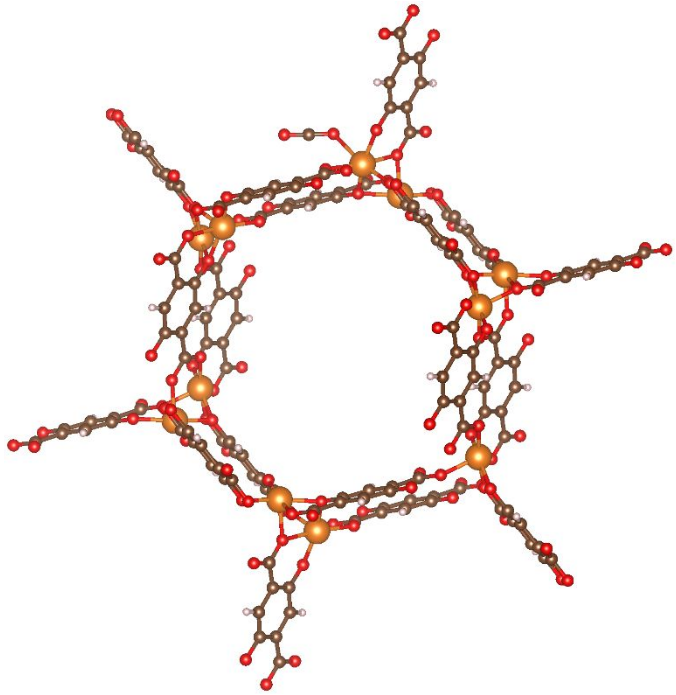

Figure S2. CO_2 E_{ads} on Mg-MOF-74.

CO_2 Adsorption on Doped Graphene (DG)

We considered substitutionally doping N, Fe, Co, Ni, and Cu in graphene as potential materials to reach our target CO_2 E_{ads}. An ionic relaxation of CO_2 in the top (Tx) and hollow (Hx) sites for each dopant is conducted to determine the most stable adsorption site (see Table S1 and Figures S3-S8). As a baseline, CO_2 adsorbed on PG is the most stable in the T site (-0.250 eV with a 3.202 Å CO_2-PG distance), which is in excellent agreement with other computational and experimental works[17–18, 30]. As PG is less stable than our target we moved to N-DG where CO_2 was placed in three different T sites (initially 1.3-2 Å N-C distance) to see if a N-C bond would form. However, there are weak interactions between N-C (of CO_2) and a chemical bond does not form. Overall, CO_2 is less stable on N-DG (-0.199 eV in hollow site) than PG and results in a shortest atomic distance of 3.108 Å (in agreement with Wang et al.)[17]. Both PG and N-DG physisorb CO_2 (primarily vdW interactions), while the transition metal dopants chemisorb CO_2. CO_2 is most stable in the H site for each of the transition metal dopants (Fe, Co, Ni, and Cu). In order of least to most energetically stable CO_2 interaction: Cu, Ni, Co, and Fe-DG result in a CO_2-dopant distance of 2.457, 2.423, 2.276, and 2.097 Å, respectively. However, the transition metal dopants interact much more strongly with CO_2 than our target CO_2 E_{ads} (see Table S1).

Table S1. CO_2 E_{ads} on PG and DG with shortest atomic distances (y (vacuum) direction for PG and elements are in parentheses if a bond is formed). Gray indicates the most stable CO_2 site.

Dopant/G/Site	E_{ads} (eV)	vdW E_{ads} (eV)	CO_2-Dopant (Å)
**PGT**	-0.250	-0.175	3.202

PGH	-0.239	-0.158	3.282
NGT1	-0.188	-0.177	3.186
NGT2	-0.188	-0.176	3.188
NGT3	-0.190	-0.177	3.179
NGTP	-0.157	-0.095	3.013
**NGH**	-0.199	-0.178	3.108
FeGT	-2.531	-0.206	2.017 (C)
FeGTP	-2.570	-0.131	2.046 (O)
**FeGH**	-2.614	-0.192	2.097 (O)
CoGT	-2.348	-0.196	4.062
**CoGH**	-2.459	-0.236	2.276 (O)
NiGT1	-2.132	-0.211	4.010
NiGT2	-2.046	-0.132	3.164
NiGH1	-2.103	-0.186	4.131
**NiGH2**	-2.198	-0.244	2.423 (O)
CuGT1	-1.384	-0.227	4.042
CuGT2	-1.334	-0.130	2.488 (O)
CuGH1	-1.364	-0.183	4.503
CuGH2	-1.431	-0.223	2.380 (O)
**CuGH3**	-1.433	-0.242	2.457 (O)

For the following figures, the odd columns are the xz plane view and the even columns are the yz plane view (unless specified otherwise). The most stable adsorption geometry is indicated by the red box.

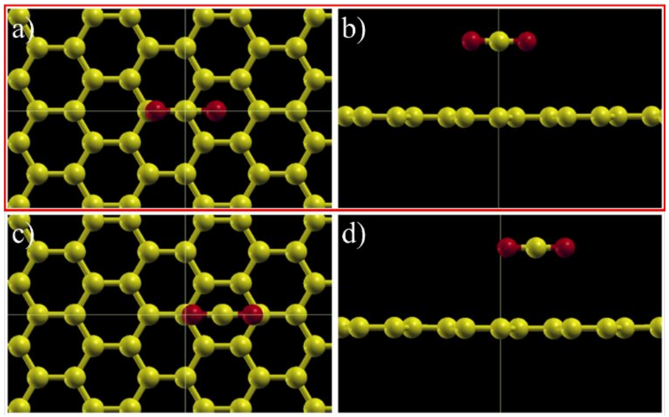

Figure S3. Ionically relaxed CO_2 configuration on PGT (a, b) and PGH (c, d) sites.

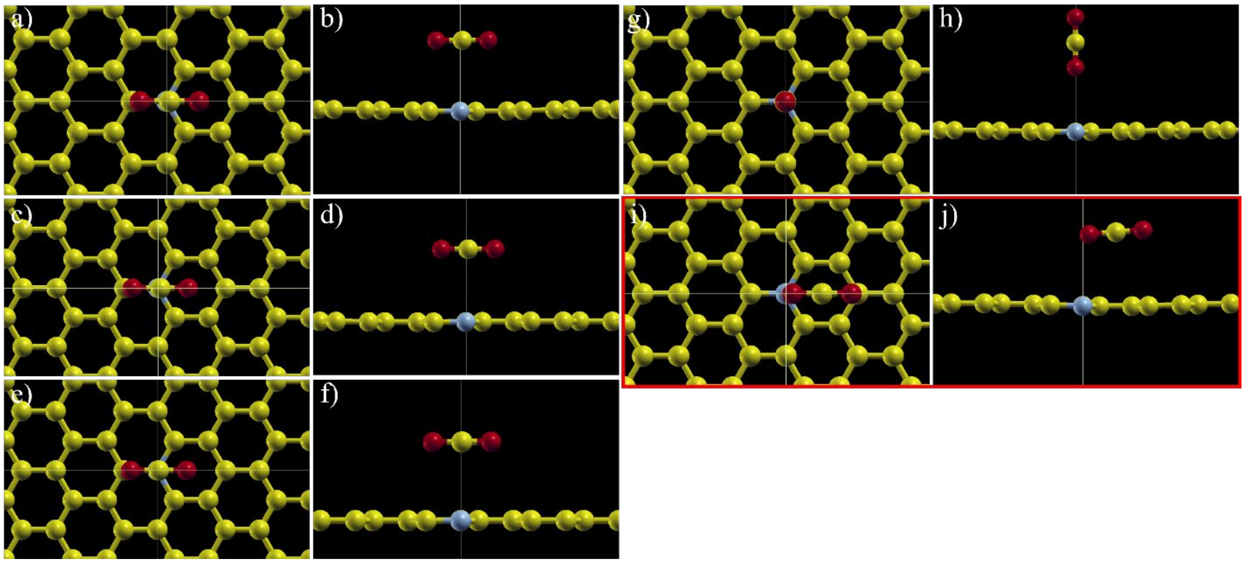

Figure S4. Ionically relaxed CO_2 configuration on NGT1 (a, b), NGT2 (c, d), NGT3 (e, f), NGTP (g, h), and NGH (i, j) sites.

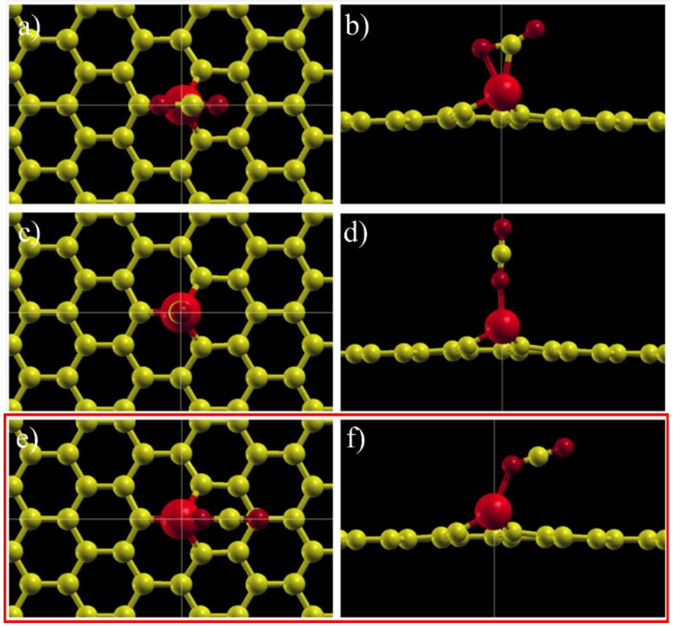

Figure S5. Ionically relaxed CO_2 configuration on FeGT (a, b), FeGTP (c, d), and FeGH (e, f) sites.

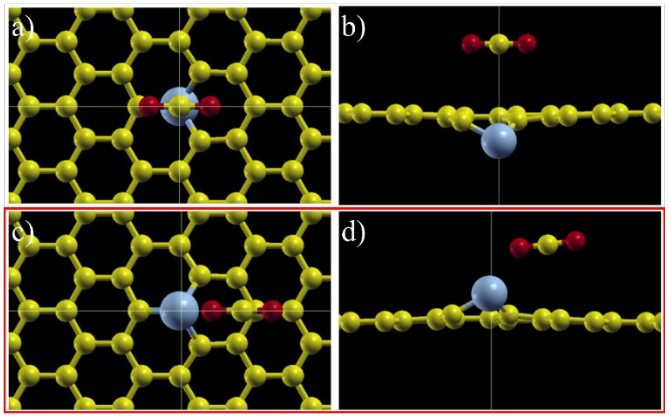

Figure S6. Ionically relaxed CO_2 configuration on CoGT (a, b) and CoGH (c, d) sites.

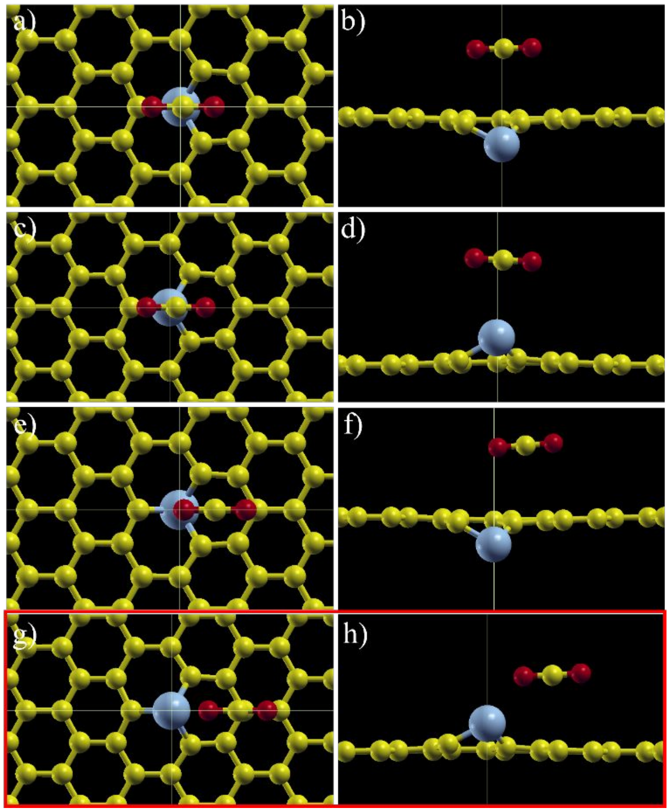

Figure S7. Ionically relaxed CO_2 configuration on NiGT1 (a, b), NiGT2 (c, d), NiGH1 (e, f), and NiGH2 (g, h) sites.

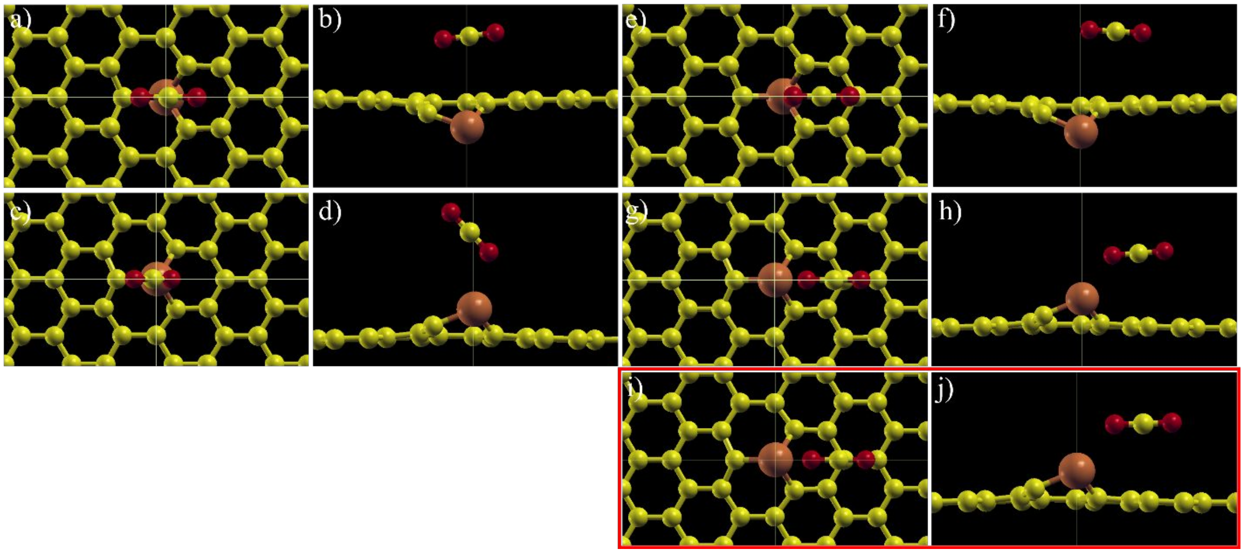

Figure S8. Ionically relaxed CO_2 configuration on CuGT1 (a, b), CuGT2 (c, d), CuGH1 (e, f), CuGH2 (g, h), and CuGH3 (i, j) sites.

CO_2 Adsorption on Functionalized Graphene (FG)

CO, OH, COOH, NH_2, NH_2CH_3, C_5H_5N, $C_6H_5NH_2$, CH_5N_3, and $C_3N_2H_4$ FMs were adsorbed on PG in the basal plane and screened as potential materials to reach our target CO_2 E_{ads} (see Table S2 and Figures S9-S19 for FMs E_{ads} and geometry; see Table S3 and Figures S20-S28 for CO_2 E_{ads} on FG and geometry). To the best of our knowledge, predicting CO, NH_2CH_3, C_5H_5N, $C_6H_5NH_2$, CH_5N_3, and $C_3N_2H_4$ adsorbing on PG and interacting with CO_2 on PG is a first of a kind. The logic of the selection has been discussed in the following three paragraphs. An ionic relaxation of each FM (and PG) in the T, H, and/or bridge (Bx) sites on PG was conducted to determine the most stable site. The H and B sites were not considered if a chemical bond was formed between the FM and PG (T site). Further, CO_2 was ionically relaxed (along with the FM and PG) on top of (Tx) and inserted (ix) next to the FM on PG to determine the most stable site.

First, we started with O-containing FMs adsorption with CO_2 on PG. CO is most stable physisorbing on PG in the T site with an E_{ads} of -0.201 eV and a 3.245 Å FM-PG distance. Then we add CO_2 into the cell, which adsorbs above CO by weak physisorption (-0.058 eV and 3.260 Å CO_2-FM distance). Next, we look at COOH FM which forms a C-C bond (1.609 Å) with PG in the T site (-0.402 eV). CO_2 is most stable physisorbing on top of COOH with an E_{ads} of -0.220 eV and a 1.911 Å O-H distance (excellent agreement with literature)[18]. We continue our search toward our target by moving to the OH FM, which forms a C-O bond (1.507 Å) with PG in the T site (-0.960 eV). CO_2 physisorbs to the OH FG in the T site with an E_{ads} of -0.152 eV and a CO_2-FM distance of 2.447 Å (in agreement with literature)[18]. However, inserting CO_2 next to OH results in the C-O bond breaking between OH and PG and forming bicarbonate with CO_2 (-0.643 eV and 1.375 Å CO_2-FM distance). In all, CO, COOH, and OH FG drive CO_2 E_{ads} farther away from our target and are not further pursued in this study.

Second, we moved to N-containing FMs in hopes of reaching our target CO_2 E_{ads}. We started with NH_2, which is most stable in the T site (-0.321 eV) on PG with the formation of a N-C bond (1.473 Å). CO_2 is then introduced into the cell and adsorbs above NH_2 with weak physisorption (-0.096 eV and 2.634 Å CO_2-FM distance), which is lower than predicted on edge FG (-0.260 eV)[18]. This is expected as CO_2 has minimal interaction with PG as it is directly above NH_2 and 5 Å above PG. However, inserting CO_2 next to NH_2 breaks the N-C bond between NH_2 and PG and forms a N-C bond (1.390 Å) with CO_2 (-0.979 eV). Since the T1 and i1 CO_2 adsorption sites on NH_2 FG are much less and more stable than our target, respectively, we transitioned to NH_2CH_3 to decrease the electronegativity (EN) difference between CO_2 and the FM. NH_2CH_3 is most stable in the T1 site with an E_{ads} of -0.265 eV and a 2.698 Å FM-PG distance. CO_2 adsorbs above NH_2CH_3 with weak physisorption (-0.078 eV and 2.776 Å CO_2-FM distance). However, the most stable CO_2 site is the i2 site, which is near our target CO_2 E_{ads} (-0.367 eV and 2.893 Å CO_2-FM distance) due to dipole-dipole interactions.

We continued our search for additional FMs to reach our target CO_2 E_{ads} and shifted to aromatic molecules. We started with C_5H_5N which is most stable in the H site due to π-π interactions (with PG) with an E_{ads} of -0.543 eV (mostly vdW interactions -0.516 eV) and a 3.228 Å FM-PG distance. Note, C_5H_5N is more stable on PG than our target CO_2 E_{ads} indicating thermal stability. Then we introduce CO_2 into the cell where CO_2 adsorbs above C_5H_5N by weak physisorption (-0.129 eV and 3.192 Å CO_2-FM distance), which is in excellent agreement with literature[31] on CO_2 interacting above C_5H_5N without PG as the vdW interactions between CO_2 and PG are minimal at 6.391 Å. For inserting CO_2, the i1 site is the most stable and is near our target CO_2 E_{ads} (-0.347 eV and 2.758 Å CO_2-FM distance) due to dipole-dipole interactions. Next, we moved to $C_6H_5NH_2$, which is most stable on PG in the H site (π-π interactions) and is more thermally stable than C_5H_5N on PG (-0.633 eV and 2.937 Å FM-PG distance). CO_2 physisorbs above $C_6H_5NH_2$ with an E_{ads} of -0.170 eV and a 2.888 Å CO_2-FM distance. Interestingly, the inserted sites cease to improve CO_2 stability by much as the i3 site is the most stable with an E_{ads} of -0.219 eV and a 3.124 Å CO_2-FM distance.

From here, we transitioned to CH_5N_3 and $C_3N_2H_4$ FMs as Lee et al.[31] predicted favorable interactions with CO_2, similar to that of C_5H_5N (without the CO_2-PG vdW energy contribution). CH_5N_3 is most stable (-0.530 eV and 2.611 Å FM-PG distance) on PG with C in the T site and the N atoms in the H sites (TH). CO_2 physisorbs on CH_5N_3 FG in the T site with an E_{ads} of -0.159 eV and a 2.734 Å CO_2-FM distance. However, a N-C bond (-0.979 eV and 1.506 Å CO_2-FM distance) was formed with CO_2 inserted next to CH_5N_3 (i3). Next, we predicted that $C_3N_2H_4$ is thermally stable on PG in the T2 site with an E_{ads} of -0.526 eV and a 3.087 Å FM-PG distance. CO_2 physisorbs on $C_3N_2H_4$ FG in the T site with an E_{ads} of -0.167 eV and a 2.883 Å CO_2-FM distance. However, similar to CH_5N_3, a N-C bond (-0.574 eV and 1.611 Å CO_2-FM distance) was formed by CO_2 in the i1 site on $C_3N_2H_4$ FG. Both CH_5N_3 and $C_3N_2H_4$ showed promise being thermally stable on PG but they interact too strongly with CO_2, indicating that Lee et al.[31] predicted local CO_2 interaction minima with CH_5N_3 and $C_3N_2H_4$. Overall, we predicted that NH_2CH_3 and C_5H_5N FG support favorable CO_2 interactions that are energetically near our target CO_2 E_{ads}. In addition, we predicted that C_5H_5N FG is thermally stable whereas NH_2CH_3 FG is not.

Table S2. FMs E_{ads} on PG with shortest atomic distances (y (vacuum) direction or elements are in parentheses if a bond is formed) in the y direction (vacuum). Gray indicates the most stable FM site.

FM/Site	E_{ads} (eV)	vdW E_{ads} (eV)	FM-PG (Å)
**CO**	-0.201	-0.116	3.245
**COOH**	-0.402	-0.298	1.609 (C)
**OH**	-0.960	-0.146	1.507 (O)
**NH_2T**	-0.321	-0.219	1.473 (N)
NH_2H	-0.231	-0.103	3.036
NH_2B	-0.211	-0.081	3.250
**NH_2CH_3T1**	-0.265	-0.193	2.698
NH_2CH_3T2	-0.265	-0.178	2.777
NH_2CH_3T3	-0.264	-0.185	2.734
NH_2CH_3T4	-0.243	-0.284	2.676
NH_2CH_3H1	-0.261	-0.182	2.758
NH_2CH_3H2	-0.261	-0.180	2.770
NH_2CH_3H3	-0.246	-0.286	2.626
NH_2CH_3B1	-0.263	-0.186	2.740
NH_2CH_3B2	-0.262	-0.165	2.830
$NHCH_3$T1	-0.317	-0.407	1.518 (N)
**$NHCH_3$T2**	-0.320	-0.403	1.516 (N)
**NH_2CH_2T**	-0.417	-0.453	1.839 (C)
C_5H_5NT	-0.494	-0.469	3.276
**C_5H_5NH**	-0.543	-0.516	3.228
C_5H_5NB	-0.542	-0.464	3.266
$C_6H_5NH_2$H1	-0.607	-0.585	3.266
**$C_6H_5NH_2$H2**	-0.633	-0.625	2.937
$C_6H_5NH_2$B	-0.618	-0.586	2.810
CH_5N_3TT	-0.493	-0.389	2.770
**CH_5N_3TH**	-0.530	-0.440	2.611
CH_5N_3HT	-0.498	-0.408	2.727
CH_5N_3HB	-0.524	-0.386	2.886
$C_3N_2H_4$T1	-0.503	-0.438	3.029
**$C_3N_2H_4$T2**	-0.526	-0.432	3.087
$C_3N_2H_4$TT1	-0.464	-0.388	3.098
$C_3N_2H_4$TT2	-0.494	-0.369	3.207
$C_3N_2H_4$TT3	-0.438	-0.395	3.080
$C_3N_2H_4$H	-0.485	-0.455	3.003

Table S3. CO_2 E_{ads} on FG with shortest atomic distances between FM and CO_2 (elements are in parentheses if a bond is formed), CO_2 and PG (y (vacuum) direction), and FM and PG (y (vacuum) direction or elements are in parentheses if a bond is formed). Gray indicates the most stable CO_2 site.

FM/G/Site	E_{ads} (eV)	vdW E_{ads} (eV)	FM-CO_2 (Å)	CO_2-PG (Å)	FM-PG (Å)
**COG**	-0.058	-0.038	3.260	6.554	3.231
**COOHG1**	-0.220	-0.074	1.911 (H-O)	4.950	1.608 (C)
COOHG2	-0.191	-0.138	2.425 (H-O)	2.810	1.608 (C)
OHGT	-0.152	-0.076	2.447	4.633	1.510 (O)
**OHGi**	-0.643	-0.137	1.375 (O-C)	2.883	3.461
NH_2GT1	-0.096	-0.082	2.634	5.056	1.475 (N)
NH_2GT2	-0.036	-0.056	2.913	5.341	1.474 (N)
**NH_2Gi1**	-0.979	-0.117	1.390 (N-C)	2.913	3.297
NH_2Gi2	-0.176	-0.199	3.134	3.001	1.474 (N)
NH_2CH_3GT	-0.078	-0.061	2.776	7.794	2.713
NH_2CH_3Gi1	-0.341	-0.219	2.928	3.220	2.793
**NH_2CH_3Gi2**	-0.367	-0.248	2.893	3.203	2.613
NH_2CH_3Gi3	-0.208	-0.168	2.942	3.219	2.616
C_5H_5NGT	-0.129	-0.126	3.192	6.391	3.199
**C_5H_5NGi1**	-0.347	-0.229	2.758	3.271	3.231
C_5H_5NGi2	-0.346	-0.233	2.719	3.208	3.200
$C_6H_5NH_2$GT1	-0.170	-0.140	2.888	6.088	2.780
$C_6H_5NH_2$GT2	-0.136	-0.135	2.967	6.080	2.806
$C_6H_5NH_2$Gi1	-0.218	-0.210	3.181	3.391	2.852
$C_6H_5NH_2$Gi2	-0.150	-0.031	2.945	3.200	2.923
**$C_6H_5NH_2$Gi3**	-0.219	-0.214	3.124	3.305	2.959
CH_5N_3GT	-0.159	-0.102	2.734	6.051	2.596
CH_5N_3Gi1	-0.449	-0.221	2.305	3.201	2.655
CH_5N_3Gi2	-0.450	-0.241	2.253	3.226	2.614
**CH_5N_3Gi3**	-0.979	-0.191	1.506 (N-C)	3.209	3.054
$C_3N_2H_4$GT	-0.167	-0.089	2.883	6.282	3.080
**$C_3N_2H_4$Gi1**	-0.574	-0.270	1.611 (N-C)	3.202	3.272
$C_3N_2H_4$Gi2	-0.571	-0.266	1.614 (N-C)	3.200	3.245

For the following figures, the odd columns are the xz plane view and the even columns are the yz plane view (unless specified otherwise). The most stable adsorption geometry is indicated by the red box.

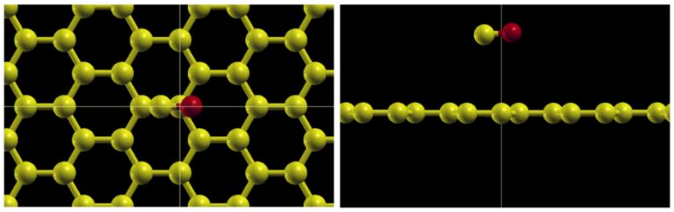

Figure S9. Ionically relaxed CO on PG.

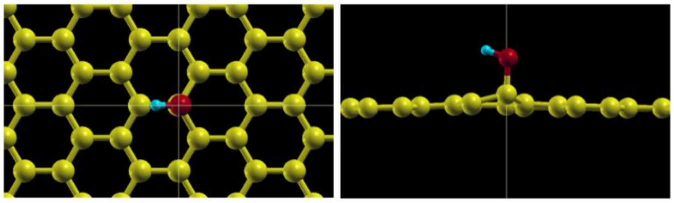

Figure S10. Ionically relaxed OH on PG.

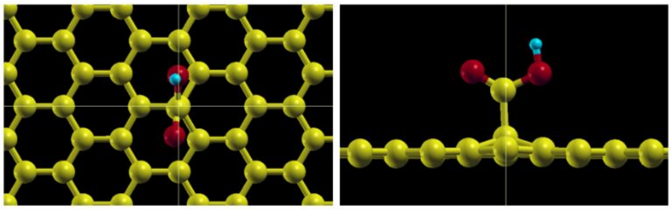

Figure S11. Ionically relaxed COOH on PG (right image is xy plane).

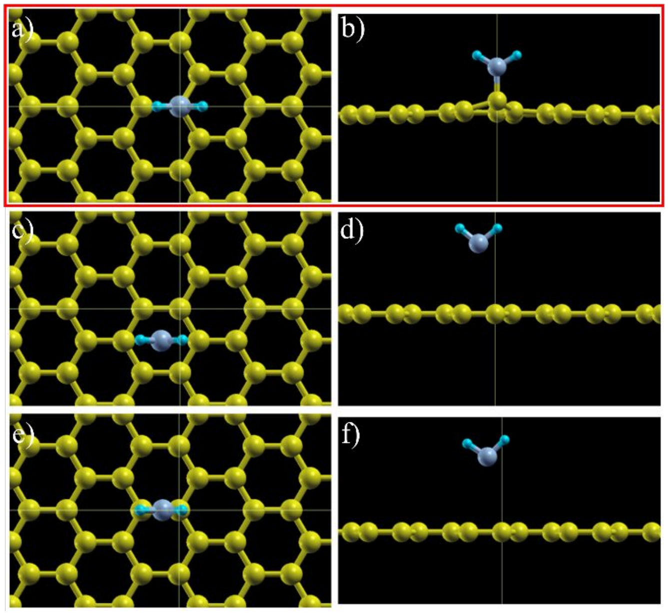

Figure S12. Ionically relaxed NH_2 configuration on PG in NH_2T (a, b), NH_2H (c, d), and NH_2B (e, f) sites.

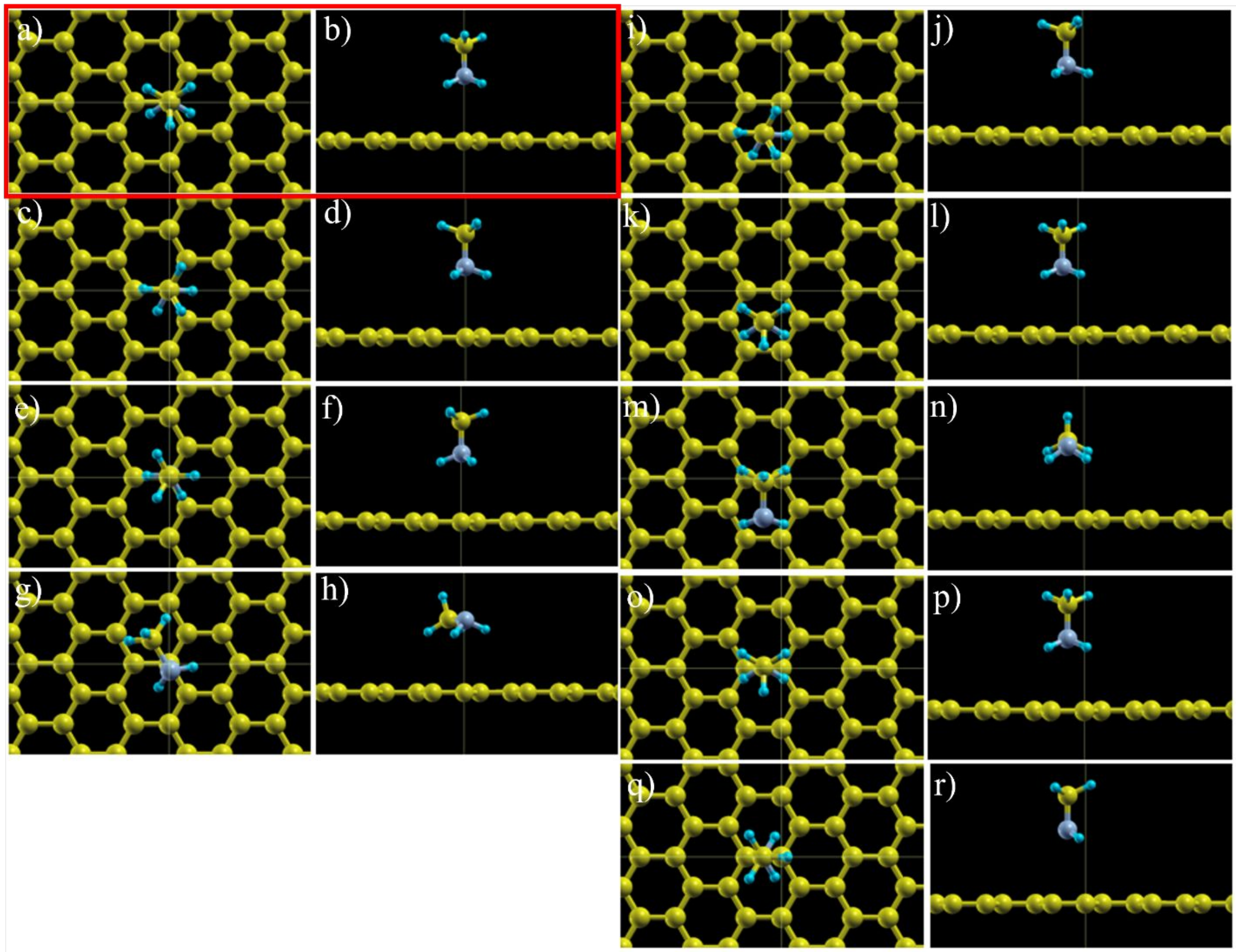

Figure S13. Ionically relaxed NH_2CH_3 configuration on PG in NH_2CH_3T1 (a, b), NH_2CH_3T2 (c, d), NH_2CH_3T3 (e, f), NH_2CH_3T4 (g, h), NH_2CH_3H1 (i, j), NH_2CH_3H2 (k, l), NH_2CH_3H3 (m, n), NH_2CH_3B1 (o, p), and NH_2CH_3B2 (q, r) sites.

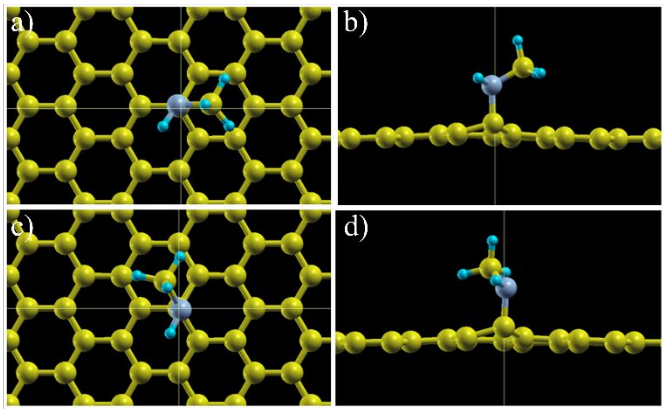

Figure S14. Ionically relaxed $NHCH_3$ configuration on PG in $NHCH_3T1$ (a, b) and $NHCH_3T2$ (c, d) sites.

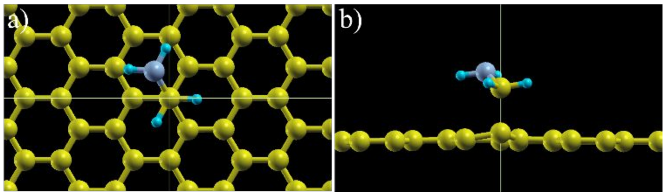

Figure S15. Ionically relaxed NH_2CH_2 configuration on PG in NH_2CH_2T (a, b) site.

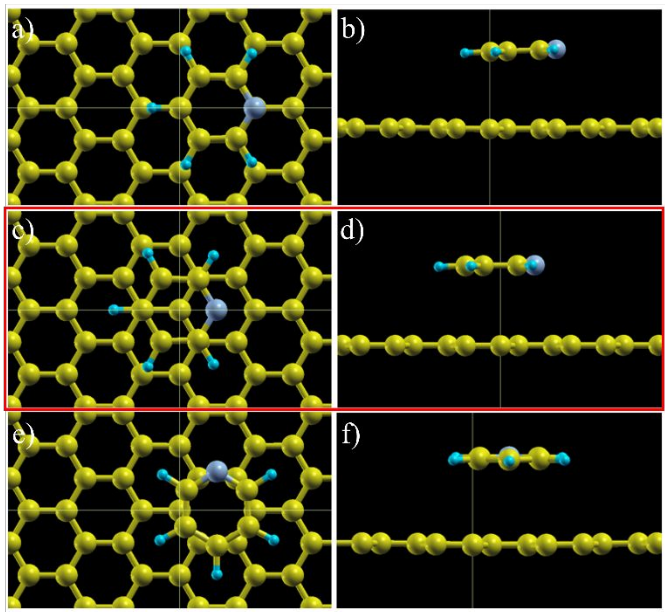

Figure S16. Ionically relaxed C_5H_5N configuration on PG in C_5H_5NT (a, b), C_5H_5NH (c, d), and C_5H_5NB (e, f) sites.

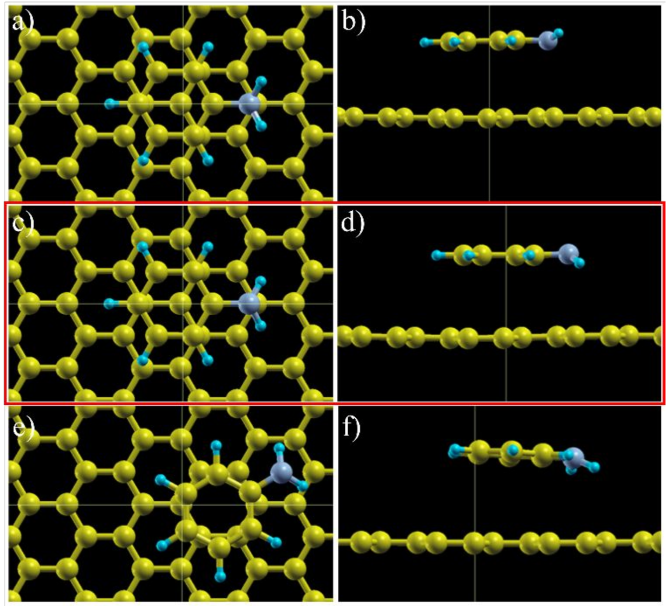

Figure S17. Ionically relaxed $C_6H_5NH_2$ configuration on PG in $C_6H_5NH_2$H1 (a, b), $C_6H_5NH_2$H2 (c, d), and $C_6H_5NH_2$B (e, f) sites.

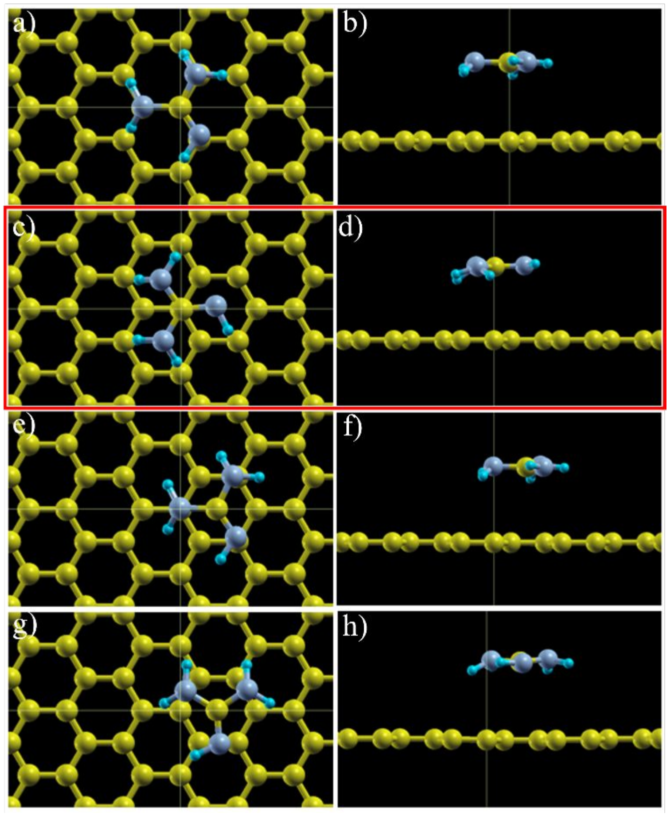

Figure S18. Ionically relaxed CH_5N_3 configuration on PG in CH_5N_3TT (a, b), CH_5N_3TH (c, d), CH_5N_3HT (e, f), and CH_5N_3HB (g, h) sites.

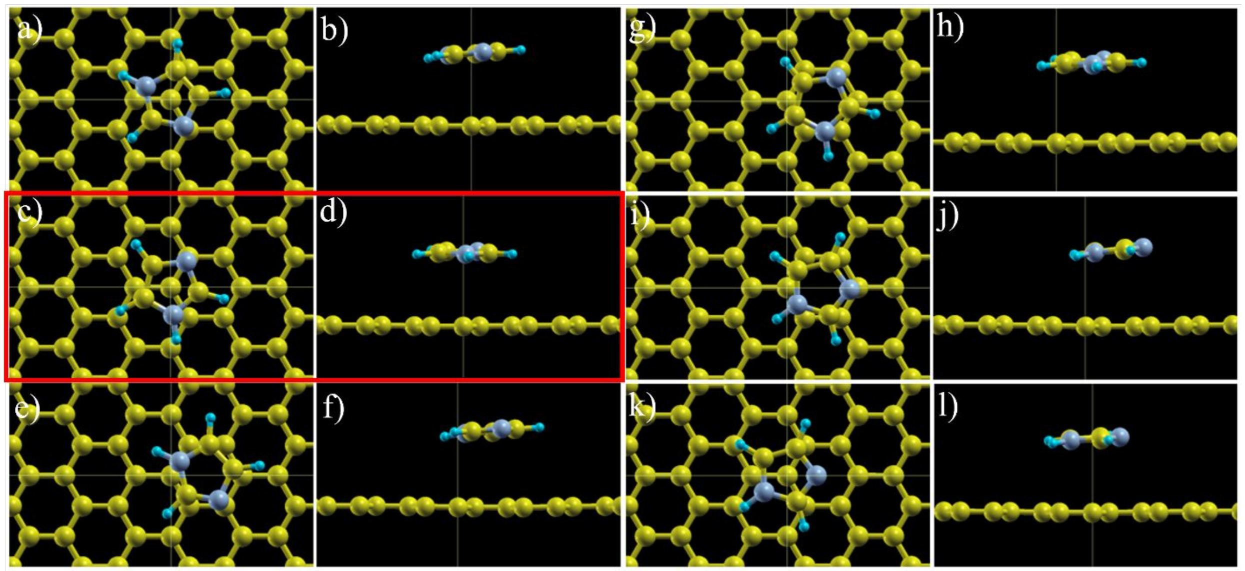

Figure S19. Ionically relaxed $C_3N_2H_4$ configuration on PG in $C_3N_2H_4$T1 (a, b), $C_3N_2H_4$T2 (c, d), $C_3N_2H_4$TT1 (e, f), $C_3N_2H_4$TT2 (g, h), $C_3N_2H_4$TT3 (i, j), and $C_3N_2H_4$H (k, l) sites.

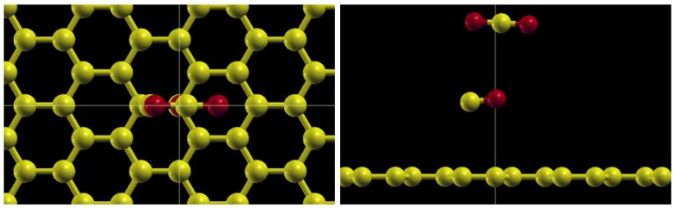

Figure S20. Ionically relaxed CO_2 configuration on COG.

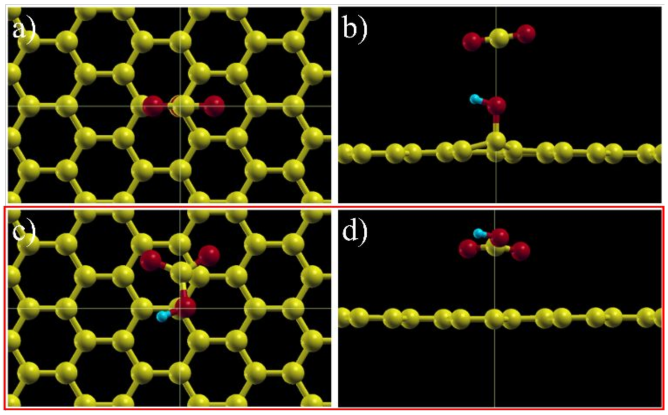

Figure S21. Ionically relaxed CO_2 configuration on OHGT (a, b) and OHGi (c, d) sites.

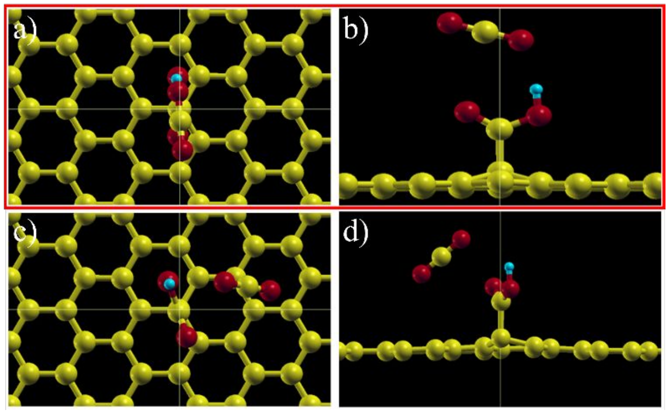

Figure S22. Ionically relaxed CO_2 configuration on COOHG1 (a, b (xy plane)) and COOHG2 (c, d) sites.

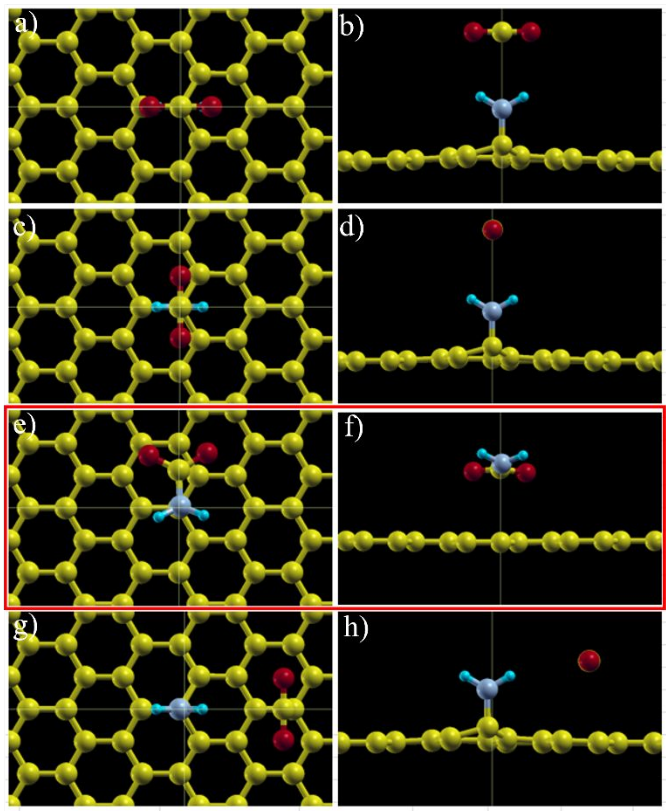

Figure S23. Ionically relaxed CO_2 configuration on NH_2GT1 (a, b), NH_2GT2 (c, d), NH_2Gi1 (e, f), and NH_2Gi2 (g, h) sites.

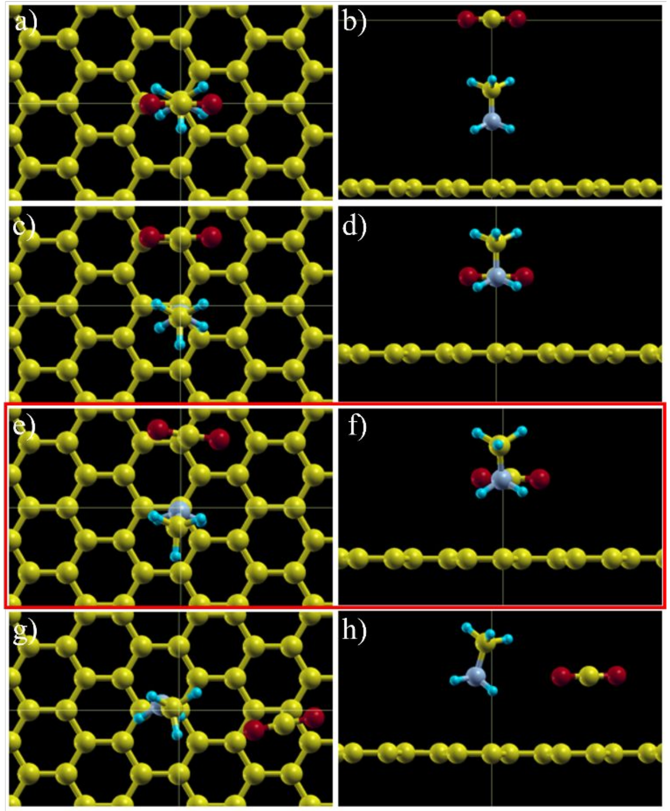

Figure S24. Ionically relaxed CO_2 configuration on NH_2CH_3GT (a, b), NH_2CH_3Gi1 (c, d), NH_2CH_3Gi2 (e, f), and NH_2CH_3Gi3 (g, h) sites.

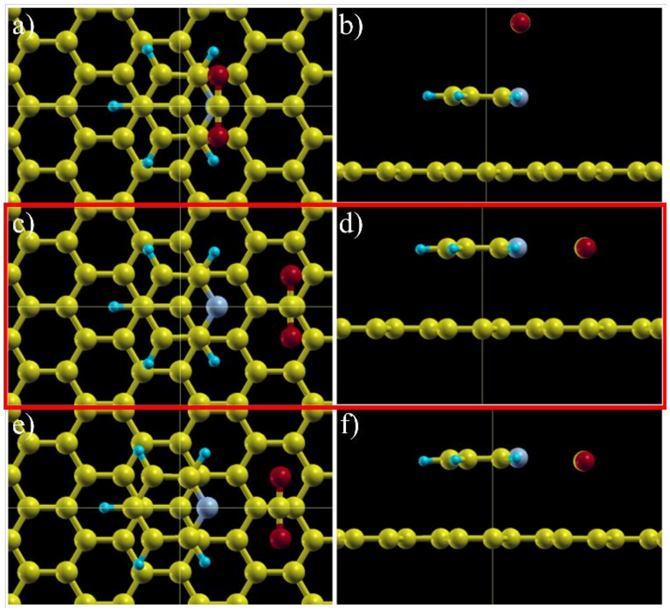

Figure S25. Ionically relaxed CO_2 configuration on C_5H_5NGT (a, b), C_5H_5NGi1 (c, d), and C_5H_5NGi2 (e, f) sites.

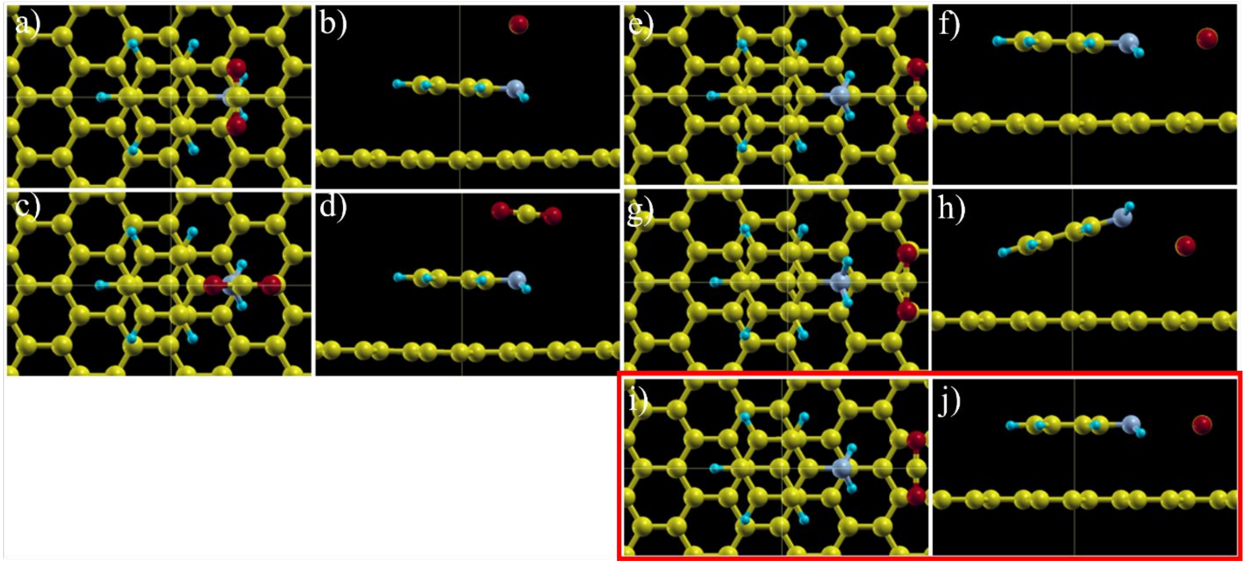

Figure S26. Ionically relaxed CO_2 configuration on $C_6H_5NH_2$GT1 (a, b), $C_6H_5NH_2$GT2 (c, d), $C_6H_5NH_2$Gi1 (e, f), $C_6H_5NH_2$Gi2 (g, h), and $C_6H_5NH_2$Gi3 (i, j) sites.

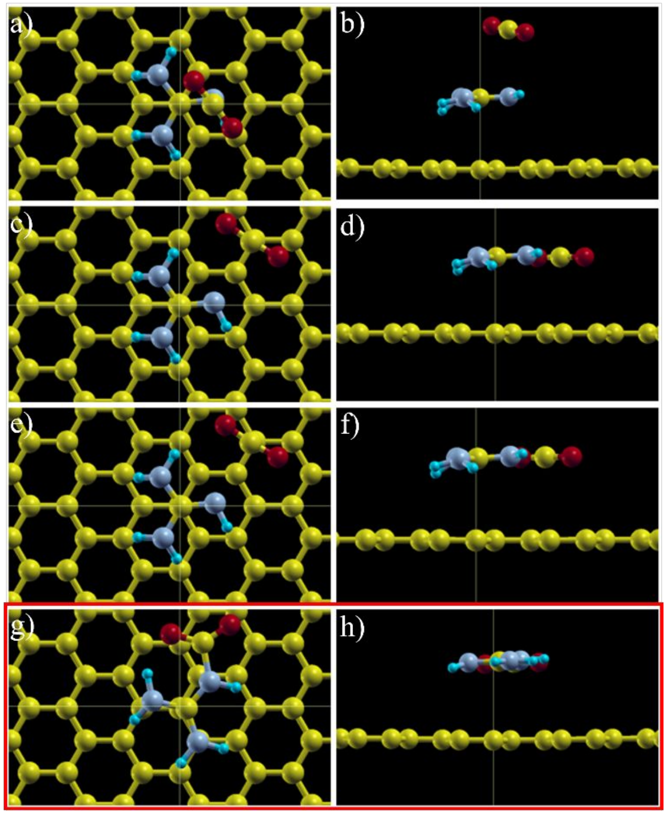

Figure S27. Ionically relaxed CO_2 configuration on CH_5N_3GT (a, b), CH_5N_3Gi1 (c, d), CH_5N_3Gi2 (e, f), and CH_5N_3Gi3 (g, h) sites.

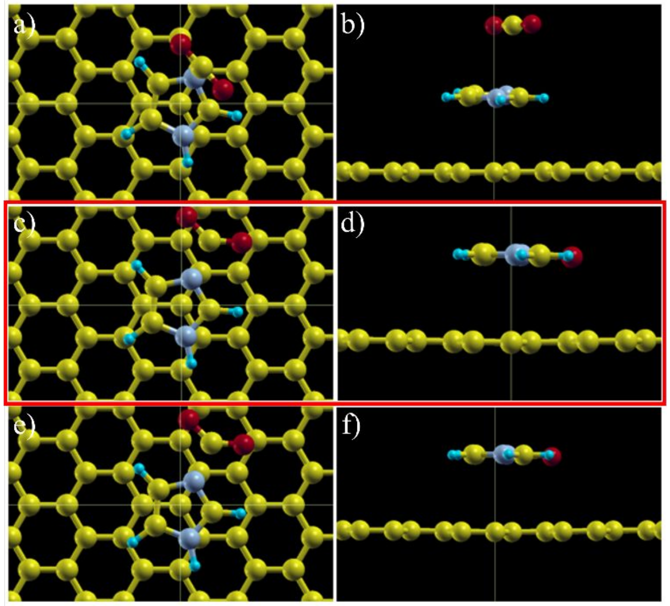

Figure S28. Ionically relaxed CO_2 configuration on $C_3N_2H_4GT$ (a, b), $C_3N_2H_4Gi1$ (c, d), and $C_3N_2H_4Gi2$ (e, f) sites.

CO_2 Adsorption on Monovacancy Defect Graphene (MG)

We predicted the defect sensitivity of methylamine and pyridine FG by calculating CO_2 E_{ads} with defects (see Table S4 and Figures S29 and S30 for FM E_{ads} and geometry; see Table S5 and Figures S31-S33 for CO_2 E_{ads} and geometry). In this study, we only considered monovacancy defects as Wang et al.[17] predicted that MG alters CO_2 E_{ads} (due to dangling bonds) greater than other common defects like Stone-Wales. For MG, CO_2 is placed in four different orientations in the defect site. The most stable site (MG4) results in a CO_2 E_{ads} of -0.370 eV and a 2.759 Å CO_2-PG distance (consistent with Wang et al.)[17]. MG provides an opportunity to increase CO_2 E_{ads} near our target, however, point defects are difficult to control and therefore methylamine and pyridine are checked to determine if they are sensitive to these defects. Methylamine physisorbs on MG with an E_{ads} of -0.380 eV and a 2.535 Å FM-MG distance. By including CO_2 in the i1 site, CO_2 is near our target CO_2 E_{ads} (-0.373 eV and 2.861 Å FM-CO_2 distance), similar to that of methylamine on PG. Pyridine forms two C-C bonds (1.520 Å, sp^3 hybridized) in the B1 site with MG (-1.875 eV), making pyridine even more thermally stable than on PG. However, pyridine is near perpendicular to MG, which results in a less stable CO_2 E_{ads} (-0.258 eV, i3), when compared to CO_2 interacting with pyridine on PG (see Table S3). Overall, methylamine is defect insensitive whereas pyridine is defect sensitive.

Table S4. FMs E_{ads} on MG with shortest atomic distances (y (vacuum) direction or elements are in parentheses if a bond is formed). Gray indicates the most stable FM site.

Material/G/Site	E_{ads} (eV)	vdW E_{ads} (eV)	FM-MG (Å)
**NH_2CH_3MG1**	-0.380	-0.191	2.535
NH_2CH_3MG2	-0.339	-0.196	2.713
C_5H_5NMGT1	-0.552	-0.436	3.202
C_5H_5NMGT2	-0.405	-0.432	3.265
C_5H_5NMGH	-0.548	-0.476	2.995
**C_5H_5NMGB1**	-1.875	-0.713	1.520 (C)
C_5H_5NMGB2	-0.886	-0.750	1.504 (C)
C_5H_5NMGB3	-1.801	-0.741	1.518 (C)

Table S5. CO_2 E_{ads} on FG on MG with shortest atomic distances between FM and CO_2 (elements are in parentheses if a bond is formed), CO_2 and PG (y (vacuum) direction), and FM and PG (y (vacuum) direction or elements are in parentheses if a bond is formed). Gray indicates the most stable CO_2 site while * indicates unstable sites.

Material/G/Site	E_{ads} (eV)	vdW E_{ads} (eV)	FM-CO_2 (Å)	CO_2-PG (Å)	FM-PG (Å)
MG1	-0.243	-0.180	-	2.763	-
MG2	-0.268	-0.179	-	2.768	-
MG3	-0.159	-0.186	-	2.826	-
**MG4**	-0.370	-0.183	-	2.759	-
**MGNH_2CH_3i1**	-0.373	-0.237	2.861	3.219	2.442
MGNH_2CH_3i2	-0.196	-0.214	2.898	3.172	2.790
MGC_5H_5NT1	-0.205	-0.148	2.678	4.463	1.521 (C)
MGC_5H_5NT2*	0.227	-0.221	1.388 (N-C)	4.008	1.522 (C)
MGC_5H_5Ni1	-0.060	-0.333	1.698 (N-C)	2.830	1.527 (C)
MGC_5H_5Ni2	-0.062	-0.328	1.714 (N-C)	2.842	1.526 (C)
**MGC_5H_5Ni3**	-0.258	-0.266	2.787	3.201	1.519 (C)

For the following figures, the odd columns are the xz plane view and the even columns are the yz plane view (unless specified otherwise). The most stable adsorption geometry is indicated by the red box.

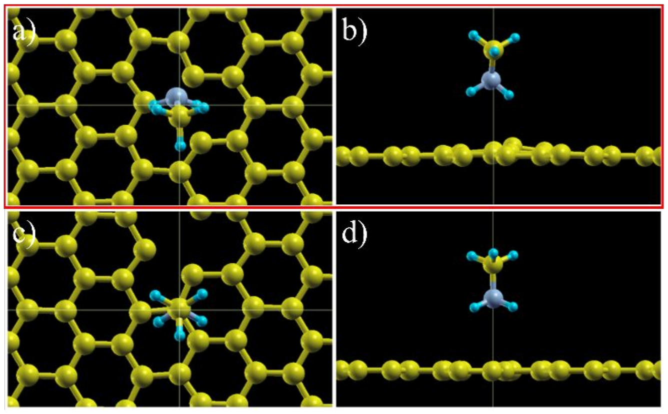

Figure S29. Ionically relaxed NH_2CH_3 configuration on MG in NH_2CH_3MG1 (a, b) and NH_2CH_3MG2 (c, d) sites.

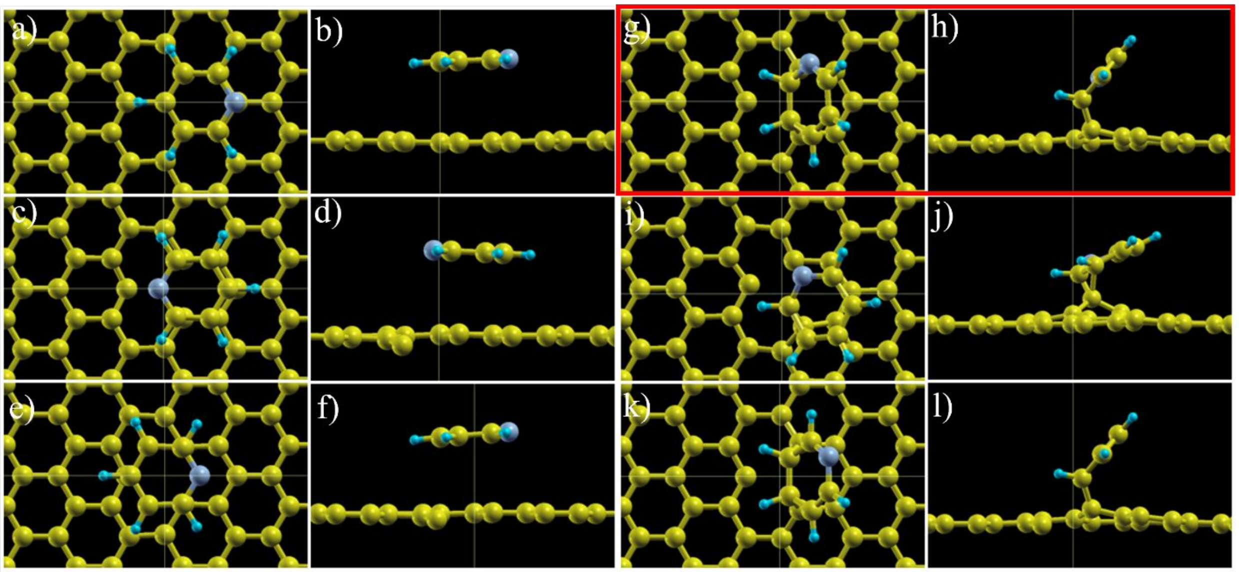

Figure S30. Ionically relaxed C_5H_5N configuration on MG in C_5H_5NMGT1 (a, b), C_5H_5NMGT2 (c, d), C_5H_5NMGH (e, f), C_5H_5NMGB1 (g, h), C_5H_5NMGB2 (i, j), and C_5H_5NMGB3 (k, l) sites.

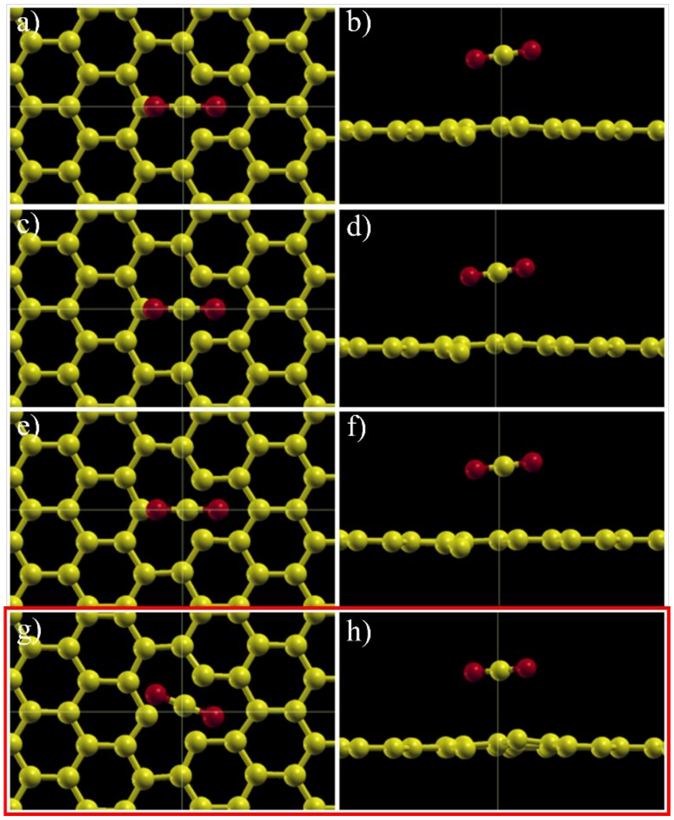

Figure S31. Ionically relaxed CO_2 configuration on MG1 (a, b), MG2 (c, d), MG3 (e, f), and MG4 (g, h) sites.

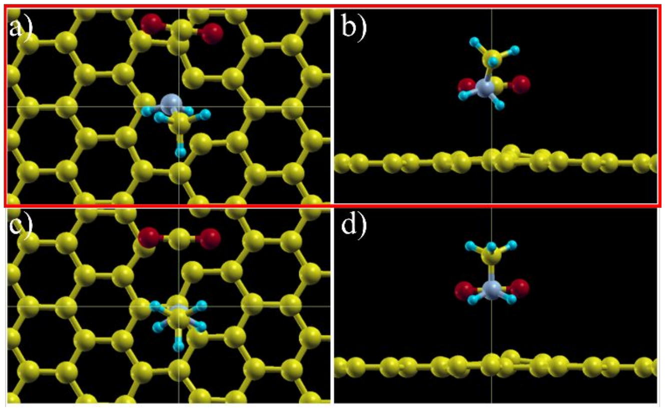

Figure S32. Ionically relaxed CO_2 configuration on MG1NH$_2$CH$_3$ (a, b) and MG2NH$_2$CH$_3$ (c, d) sites.

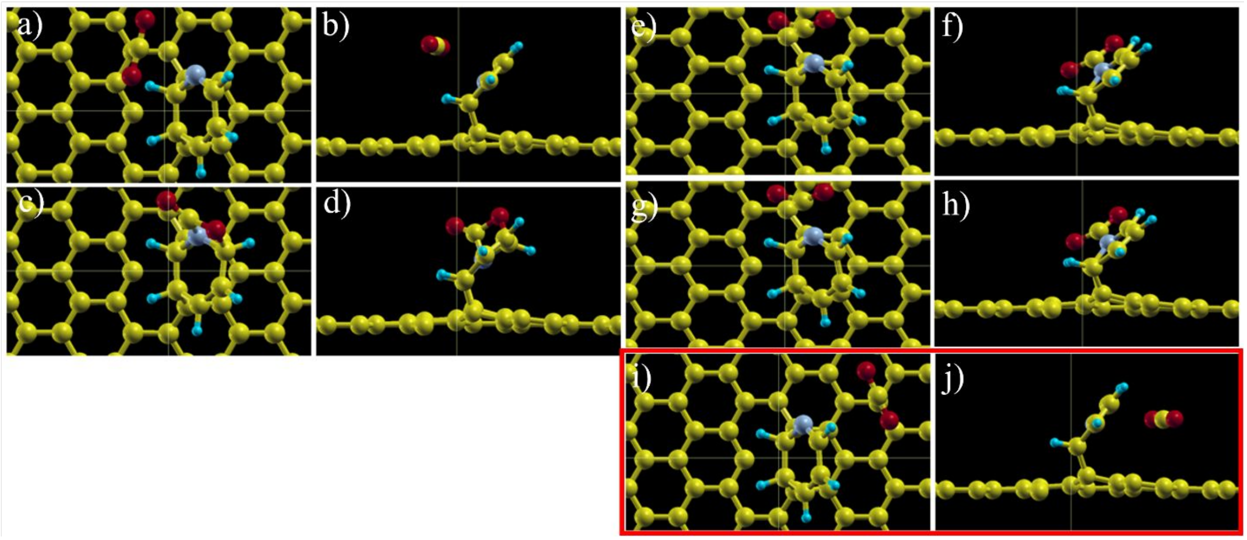

Figure S33. Ionically relaxed CO_2 configuration on MGC_5H_5NT1 (a, b), $MGC_5H_5NT2^*$ (c, d), MGC_5H_5Ni1 (e, f), MGC_5H_5Ni2 (g, h), and MGC_5H_5Ni3 (i, j) sites.

Selectivity with E_{ads}

While selectivity will be dependent on sticking coefficient, kinetic diameter, and pore size of the material in the realistic carbon capture setting, the comparative E_{ads} of CO_2 is a necessary parameter to investigate at the DFT level (see Table S6 and Figures S34-S42). H_2O, O_2, and N_2 all physisorb on PG (see Figures S34, S37, and S40) and are most stable in the H site with E_{ads} of -0.188, -0.206, and -0.208 eV, respectively. PG energetically favors CO_2 (-0.250 eV) over H_2O, O_2, and N_2, but is most competitive with O_2 and N_2. However, O_2 and N_2 prefer to adsorb onto PG rather than methylamine and pyridine FG (see Table S6), leaving the i sites open for CO_2. Nevertheless, H_2O is more stable than CO_2 on methylamine and pyridine FG due to N-H bonding in the i1 sites (see Figures S35 and S36). H_2O forms a N-H bond with both methylamine and pyridine with E_{ads} of -0.557 eV and -0.482 eV, respectively.

Table S6. N_2, O_2, and H_2O E_{ads} on FG with shortest atomic distances between FM and gas (elements are in parentheses if a bond is formed), gas and PG (y (vacuum) direction), and FM and PG (y (vacuum) direction or elements are in parentheses if a bond is formed). Gray indicates the most stable gas molecule site while * indicates unstable sites.

Gas/G/FM/Site	E_{ads} (eV)	vdW E_{ads} (eV)	FM-Gas (Å)	Gas-PG (Å)	FM-PG (Å)
H_2OGT	-0.175	-0.077	-	3.144	-
**H_2OGH**	-0.188	-0.082	-	3.072	-
H_2OGB	-0.178	-0.077	-	3.143	-
$H_2OGNH_2CH_3T$	-0.052	-0.020	2.859	7.880	2.732

**$H_2OGNH_2CH_3i$ 1**	-0.557	-0.115	1.823 (N-H)	3.275	2.677
$H_2OGNH_2CH_3i2$	-0.490	-0.142	1.843 (N-H)	2.544	2.598
$H_2OGC_5H_5NT1$	-0.284	0.025	1.846 (N-H)	4.885	2.817
$H_2OGC_5H_5NT2$	-0.479	-0.149	1.829 (N-H)	3.190	3.238
**$H_2OGC_5H_5Ni1$**	-0.482	-0.147	1.826 (N-H)	3.011	3.221
$H_2OGC_5H_5Ni2$	-0.462	-0.147	1.851 (N-H)	2.556	3.261
O_2GT	-0.195	-0.105	-	3.077	-
**O_2GH**	-0.206	-0.116	-	3.027	-
O_2GB	-0.191	-0.110	-	3.072	-
$O_2GNH_2CH_3T$	-0.054	-0.016	2.936	7.974	2.770
$O_2GNH_2CH_3i1$	-0.088	-0.117	3.476	3.186	2.719
**$O_2GNH_2CH_3i2$**	-0.156	-0.110	2.600	3.168	2.752
$O_2GC_5H_5NT1$	-0.058	-0.030	2.954	6.340	3.161
$O_2GC_5H_5NT2$*	2.451	-0.062	1.469 (C-O)	5.063	2.894
$O_2GC_5H_5Ni1$*	0.172	0.192	2.723	2.943	4.647
**$O_2GC_5H_5Ni2$**	-0.146	-0.102	2.553	3.163	3.198
N_2GT1	-0.199	-0.121	-	3.189	-
N_2GT2	-0.168	-0.081	-	3.260	-
**N_2GH1**	-0.208	-0.135	-	3.163	-
N_2GH2	-0.179	-0.097	-	3.083	-
N_2GB	-0.203	-0.123	-	3.245	-
$N_2GNH_2CH_3T$	-0.051	-0.049	2.919	7.881	2.690
**$N_2GNH_2CH_3i1$**	-0.189	-0.159	3.114	3.330	2.591
$N_2GNH_2CH_3i2$	-0.151	-0.128	2.875	3.188	2.776
$N_2GC_5H_5NT1$	-0.063	-0.051	3.137	6.331	3.064
$N_2GC_5H_5NT2$	-0.083	-0.090	3.267	6.471	3.134
$N_2GC_5H_5Ni1$*	0.186	0.209	2.775	3.108	4.820
**$N_2GC_5H_5Ni2$**	-0.174	-0.118	2.657	3.309	3.201

For the following figures, the odd columns are the xz plane view and the even columns are the yz plane view (unless specified otherwise). The most stable adsorption geometry is indicated by the red box.

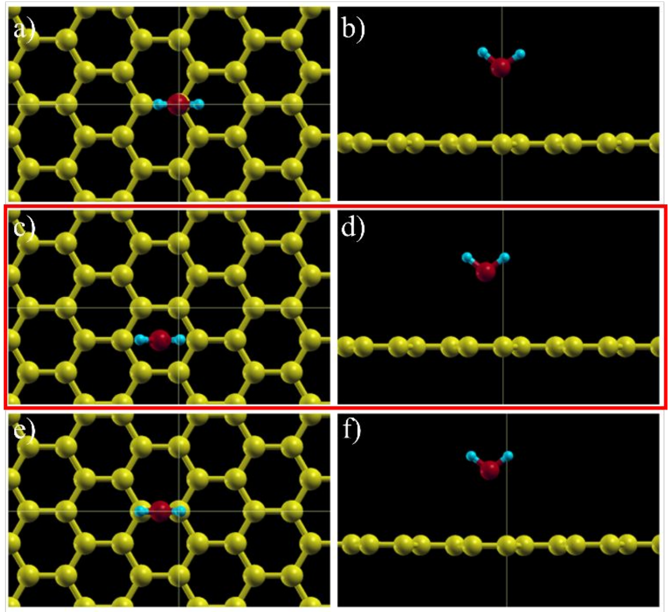

Figure S34. Ionically relaxed H_2O configuration on PG in H_2OGT (a, b), H_2OGH (c, d), and H_2OGB (e, f) sites.

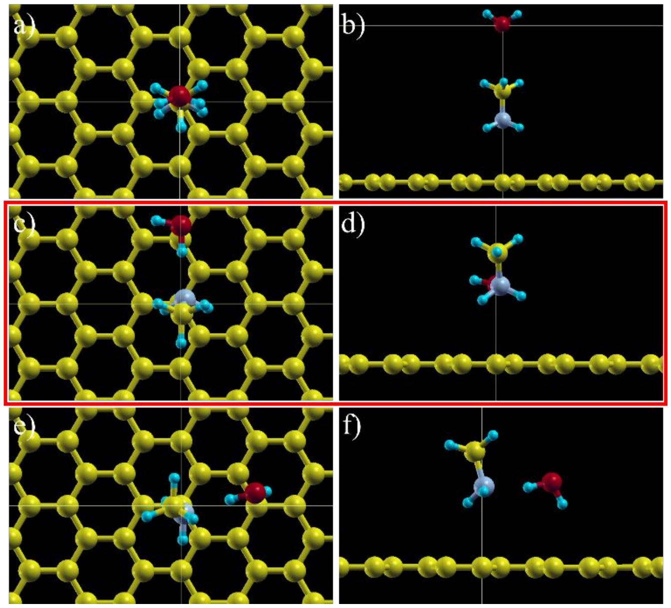

Figure S35. Ionically relaxed H_2O configuration on GNH_2CH_3 in $H_2OGNH_2CH_3T$ (a, b), $H_2OGNH_2CH_3i1$ (c, d), and $H_2OGNH_2CH_3i2$ (e, f) sites.

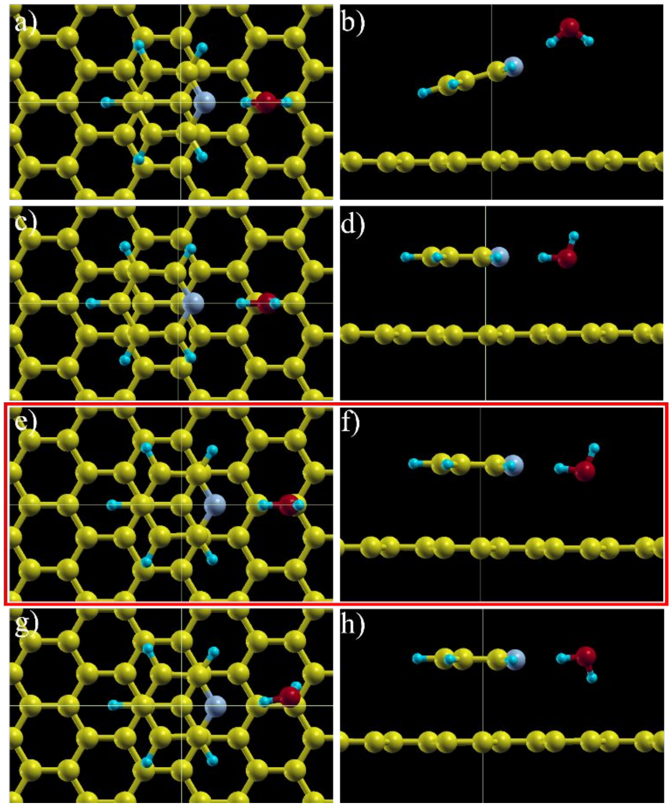

Figure S36. Ionically relaxed H_2O configuration on GC_5H_5N in $H_2OGC_5H_5NT1$ (a, b), $H_2OGC_5H_5NT1$ (c, d), $H_2OGC_5H_5Ni1$ (e, f), and $H_2OGC_5H_5Ni2$ (g, h) sites.

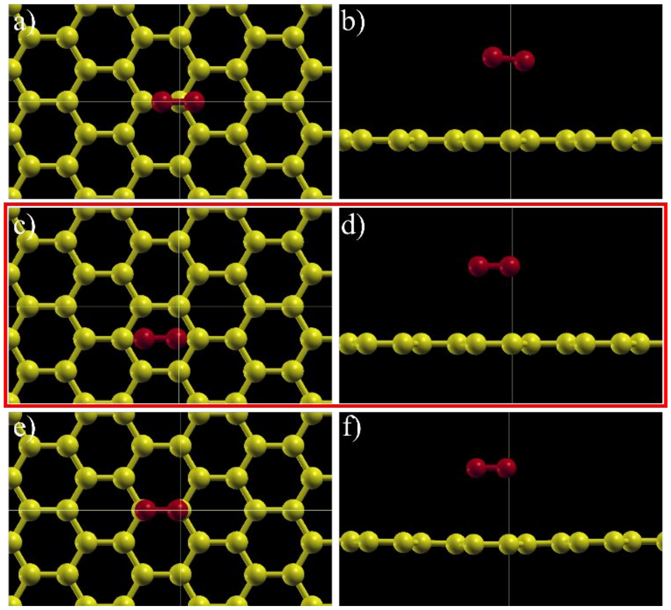

Figure S37. Ionically relaxed O_2 configuration on PG in O_2GT (a, b), O_2GH (c, d), and O_2GB (e, f) sites.

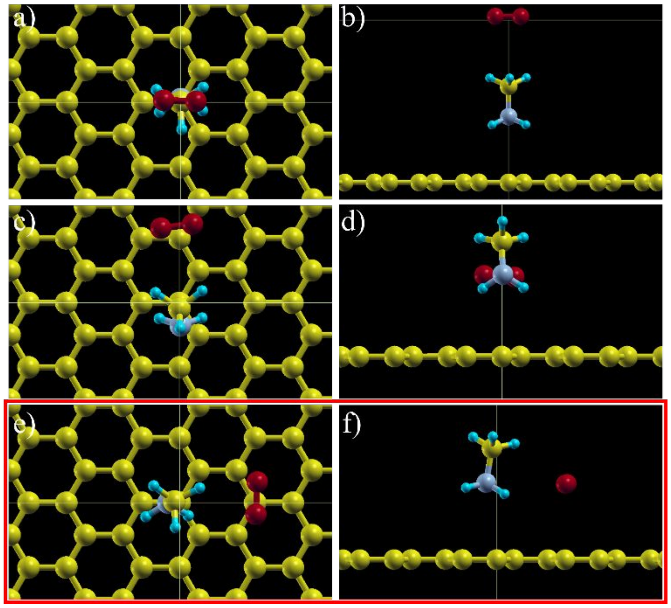

Figure S38. Ionically relaxed O_2 configuration on GNH_2CH_3 in $O_2GNH_2CH_3T$ (a, b), $O_2GNH_2CH_3i1$ (c, d), and $O_2GNH_2CH_3i2$ (e, f) sites.

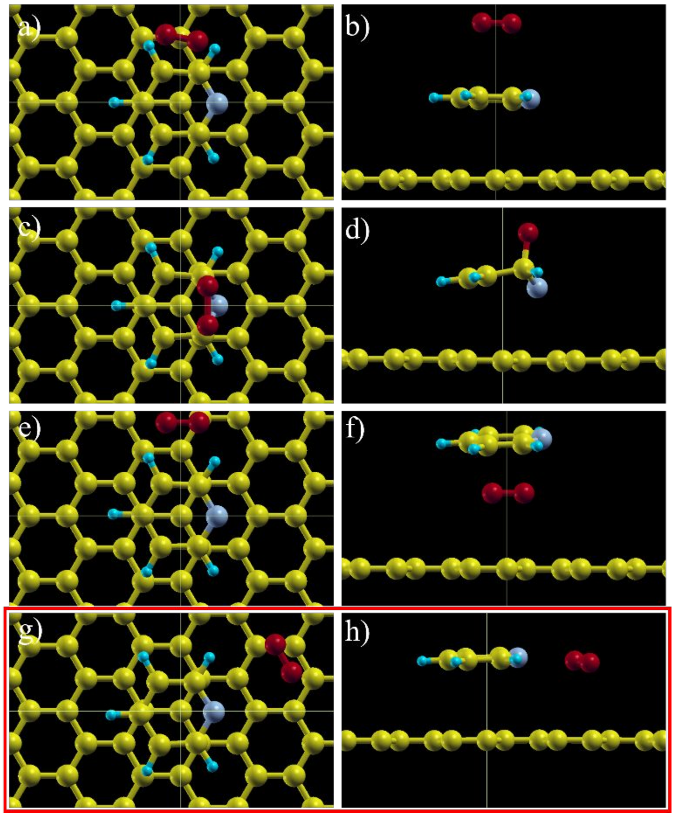

Figure S39. Ionically relaxed O_2 configuration on GC_5H_5N in $O_2GC_5H_5NT1$ (a, b), $O_2GC_5H_5NT2$* (c, d), $O_2GC_5H_5Ni1$* (e, f), and $O_2GC_5H_5Ni2$ (g, h) sites. * indicates stability.

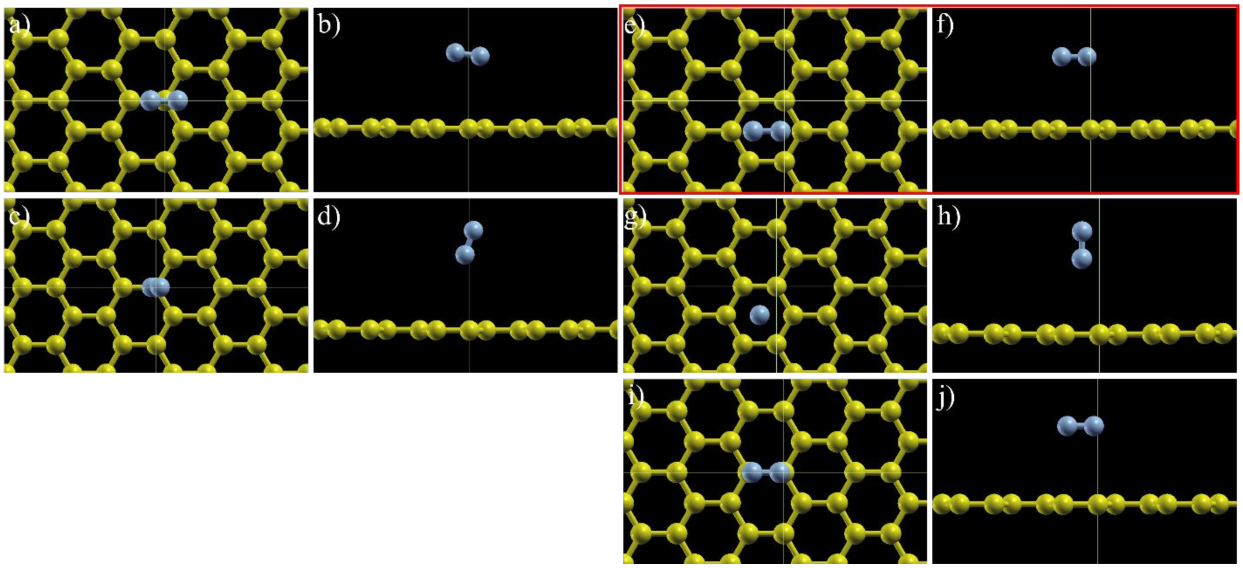

Figure S40. Ionically relaxed N_2 configuration on PG in N_2GT1 (a, b), N_2GT2 (c, d), N_2GH1 (e, f), N_2GH2 (g, h), and N_2GB (i, j) sites.

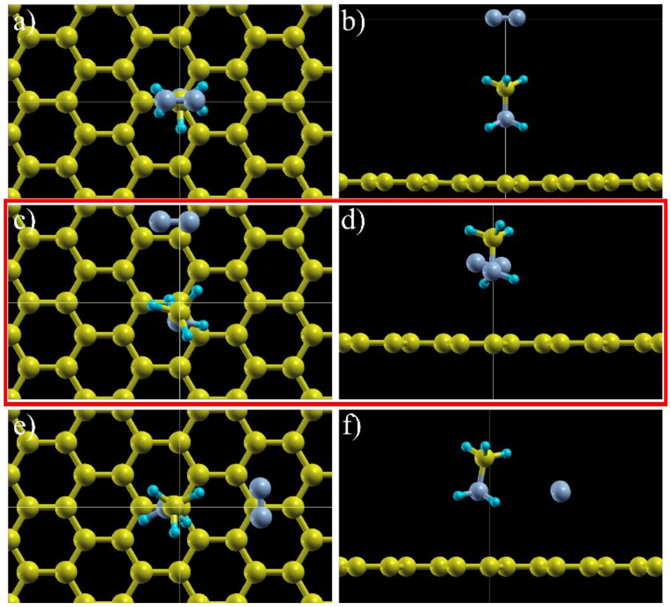

Figure S41. Ionically relaxed N_2 configuration on GNH_2CH_3 in $N_2GNH_2CH_3T$ (a, b), $N_2GNH_2CH_3i1$ (c, d), and $N_2GNH_2CH_3i2$ (e, f) sites.

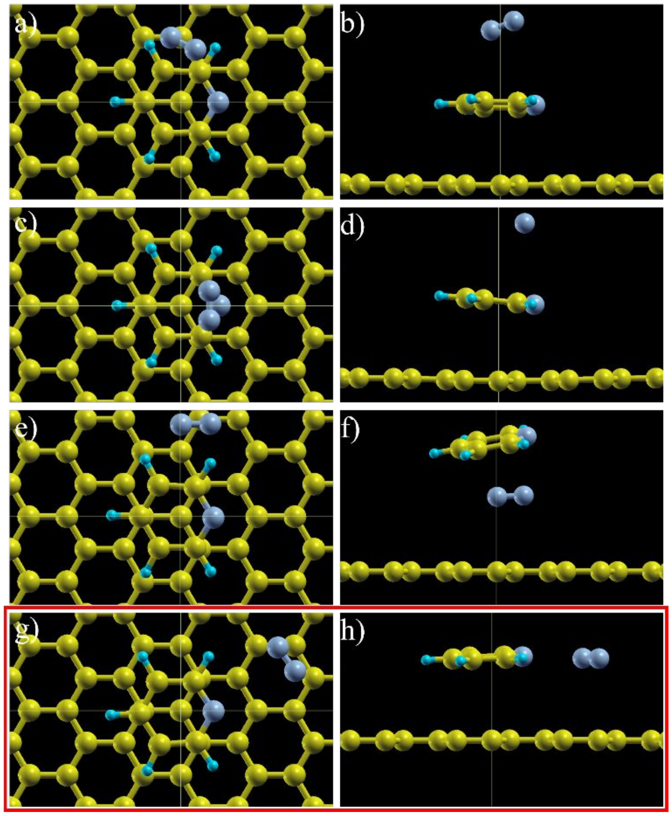

Figure S42. Ionically relaxed N_2 configuration on GC_5H_5N in $N_2GC_5H_5NT1$ (a, b), $N_2GC_5H_5NT1$ (c, d), $N_2GC_5H_5Ni1$* (e, f), and $N_2GC_5H_5Ni2$ (g, h) sites. * indicates stability.

Coadsorption with E_{ads}

Pyridine and methylamine FG energetically favor H_2O over CO_2, which opens the door for either cooperative or competitive adsorption (see Table S7 and Figures S43-S45). For methylamine FG, CO_2 forms carbonic acid with H_2O, which then dissociates H to NH_2CH_3 and forms $NH_3CH_3^+$ and bicarbonate (see Figure S43). The CO_2 E_{ads} on H_2O adsorbed methylamine FG is -0.472 eV, while the bicarbonate E_{ads} on $NH_3CH_3^+$ FG is -0.892 eV. However, both bicarbonate and $NH_3CH_3^+$ will desorb together as their E_{ads} on PG is the least stable (-0.465 eV). For pyridine FG, CO_2 physisorbs to $H_2OGC_5H_5N$ with an E_{ads} of -0.282 eV and fails to form carbonic acid or bicarbonate with H_2O (see Figure S44). However, carbonic acid's E_{ads} is -0.943 eV on pyridine FG (see Figure S45) due to N-H bond (1.577 Å). Similar to methylamine FG, pyridine and carbonic acid will desorb together as their E_{ads} on PG (-0.771 eV) is less than carbonic acid separating from pyridine FG. Altogether, both methylamine and pyridine FG support cooperative adsorption with H_2O and CO_2, and both FMs desorb with bicarbonate and carbonic acid, respectively.

Table S7. CO_2 E_{ads} on H_2O adsorbed FG with shortest atomic distances between FM and gas (elements are in parentheses if a bond is formed), gas and PG (y (vacuum) direction), and FM and

PG (y (vacuum) direction or elements are in parentheses if a bond is formed). Gray indicates the most stable gas molecule site while * indicates unstable sites.

X:Y/G/FM/Site	E_{ads} (eV)	vdW E_{ads} (eV)	H_2O-CO_2 (Å)	FM-CO_2 (Å)	FM-H_2O (Å)	CO_2-PG (Å)	H_2O-PG (Å)	FM-PG (Å)
**CO_2:$H_2OGNH_2CH_3i$**	-0.472	-0.283	1.434 (O-C)	1.650 (H-O)	2.113	3.136	3.023	2.922
$HCO_3GNH_3CH_3$	-0.892	-0.262						
$HCO_3NH_3CH_3G$	-0.465	-0.422						
CO_2:$H_2OGC_5H_5NT$	-0.201	-0.093	2.724	3.758	1.797 (N-H)	5.794	2.517	3.200
**CO_2:$H_2OGC_5H_5Ni1$**	-0.282	-0.116	2.772	5.158	1.841 (N-H)	3.404	2.379	3.279
CO_2:$H_2OGC_5H_5Ni2$	-0.275	-0.097	2.760	5.221	1.841 (N-H)	3.632	2.395	3.242
CO_2:$H_2OGC_5H_5Ni3$	-0.253	-0.107	2.740	4.575	1.825 (N-H)	4.098	2.446	3.231
CO_2:$H_2OGC_5H_5Ni4$	-0.239	-0.089	2.711	4.510	1.831 (N-H)	4.410	2.419	3.241
**$H_2CO_3GC_5H_5Ni$**	-0.943	-0.352	-	-	1.577 (N-H)	-	3.131	3.263
$H_2CO_3C_5H_5NG$	-0.771	-0.765						

For the following figures, the odd columns are the xz plane view and the even columns are the yz plane view (unless specified otherwise). The most stable adsorption geometry is indicated by the red box.

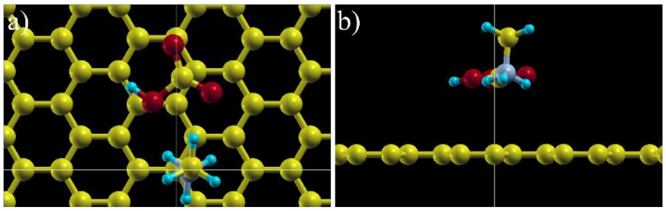

Figure S43. Ionically relaxed CO_2 configuration on $H_2OGNH_2CH_3$ in CO_2:$H_2OGNH_2CH_3i$ (a, b) site.

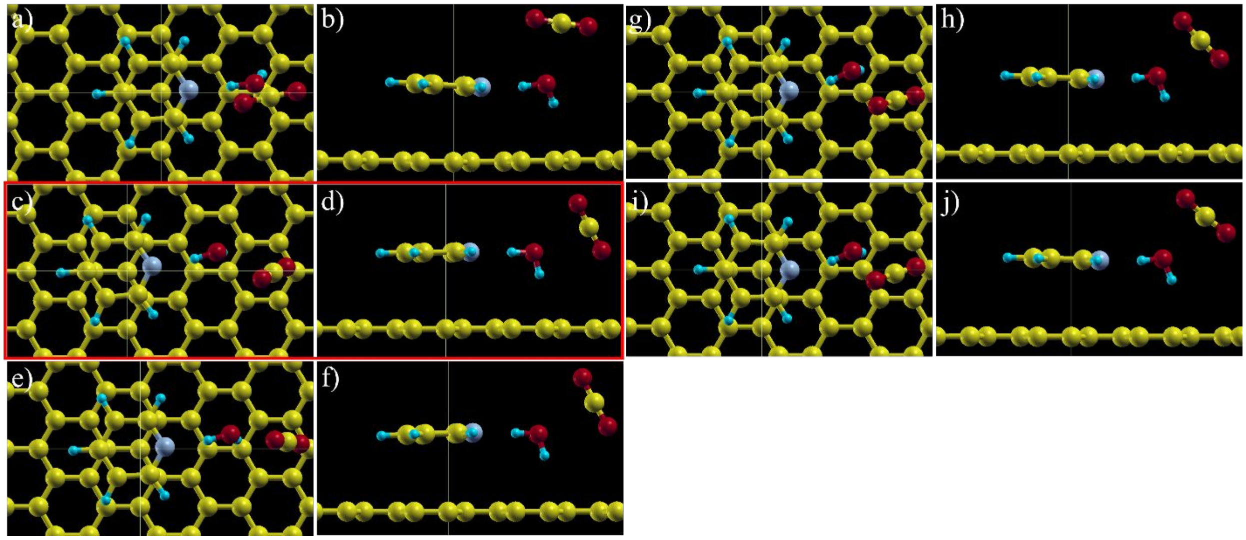

Figure S44. Ionically relaxed CO_2 configuration on $H_2OGC_5H_5N$ in CO_2:$H_2OGC_5H_5NT$ (a, b), CO_2:$H_2OGC_5H_5Ni1$ (c, d), CO_2:$H_2OGC_5H_5Ni2$ (e, f), CO_2:$H_2OGC_5H_5Ni3$ (g, h), and CO_2:$H_2OGC_5H_5Ni4$ (i, j) sites.

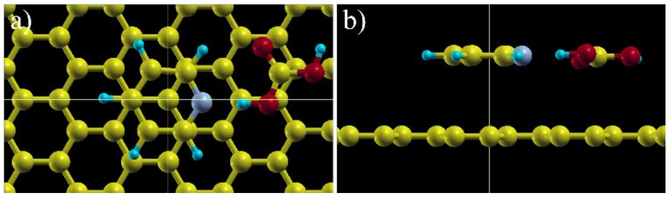

Figure S45. Ionically relaxed H_2CO_3 configuration on GC_5H_5N in $H_2CO_3GC_5H_5Ni$ (a, b) site.

Coverage Effects on CO_2 E_{ads}

Coverage of CO_2 on graphene ceases to play a significant role according to CO_2 E_{ads}. To procure this discovery, we used the Langmuir Isotherm[32,33] model to predict CO_2 coverage at select CO_2 concentrations (see equation S1).

$$\theta = \frac{KP}{1 + KP} \tag{S1}$$

Where θ is coverage (CO_2 molecules/C atom), K is the Langmuir constant (1.12 bar^{-1} for CO_2)[34], and P is partial pressure of the gas (bar). At 400 ppm, CO_2 has a graphene surface coverage of 1.60 x 10^{-18} g/CO_2 molecule,[34] which is ~2211 C atoms/CO_2 molecule for PG. The computational limit for standard plane-wave DFT code is around a couple hundred atoms. We

incrementally calculated CO_2 E_{ads} on PG in sheets of 36, 252, 780, and 1152 C atoms, which required the use of real-space DFT at the PBE-D2 level of theory to account for the large graphene sheets (see Table S8 and Figure S46). ARES[35] is a real-space pseudopotential electronic structure calculation package based on DFT, with highly parallelizable features making the simulation of our system possible. For validation, the real-space DFT (36 C atom CO_2 E_{ads}) calculation is consistent with our exemplary QE calculation at the same level of theory (see Table S1). CO_2 E_{ads} is well-converged for all PG sheet sizes indicating that enthalpy alone does not play a role in coverage. Furthermore, a calculation at a CO_2 concentration of 400 ppm (2211 C atoms/CO_2 molecule) is unnecessary. In addition, we calculated the CO_2 E_{ads} on methylamine and pyridine FG at select coverages. Van der Waals interactions are minimal at 7 Å and negligible >10 Å, corresponding to 60 and 96 C atoms/CO_2 molecule, respectively. CO_2 E_{ads} converges for both methylamine and pyridine FG at a coverage of 60 C atoms/CO_2 molecule (15000 ppm) and lower as the shortest atomic distances between repeating images are ~7 Å. Interestingly, CO_2 is more stable on the 36 C atom sheet (than 60 and 96) of methylamine FG due to O-H interaction of CO_2 and methylamine in repeating images (see Figure S47). To the contrary, CO_2 is less stable on the 36 C atom sheet (than 60 and 96) of pyridine FG due to the H-H interactions of pyridine in repeating images (see Figure S48). Indicating that a higher methylamine coverage (>15000 ppm) and a lower pyridine coverage (<15000 ppm) is preferred for CO_2 adsorption.

Table S8. CO_2 E_{ads} (eV) on PG, NH_2CH_3-FG, and C_5H_5N-FG with respect to coverage

CO_2 concentration (ppm)	25000	15000	9300	3520	1135	768
# C atoms/CO_2 molecule	36	60	96	252	780	1152
PG (ARES)	-0.201	-	-	-0.194	-0.196	-0.192
NH_2CH_3-FG (QE)	-0.357	-0.322	-0.322	-	-	-
C_5H_5N-FG (QE)	-0.338	-0.352	-0.349	-	-	-

For figures S46-S48, the PG and FG images are in the xz plane view.

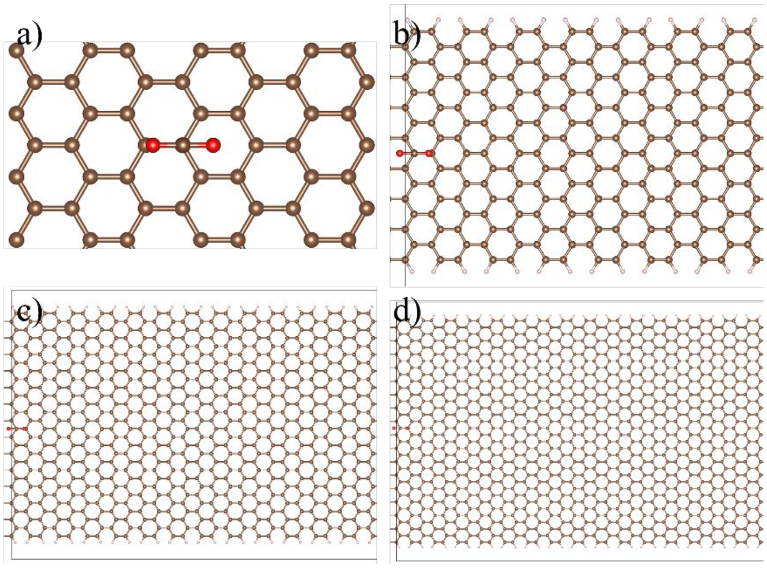

Figure S46. Single point configuration of CO_2 on PG in 36 (a), 252 (b), 780 (c), and 1152 (d) C atom sheets using ARES.

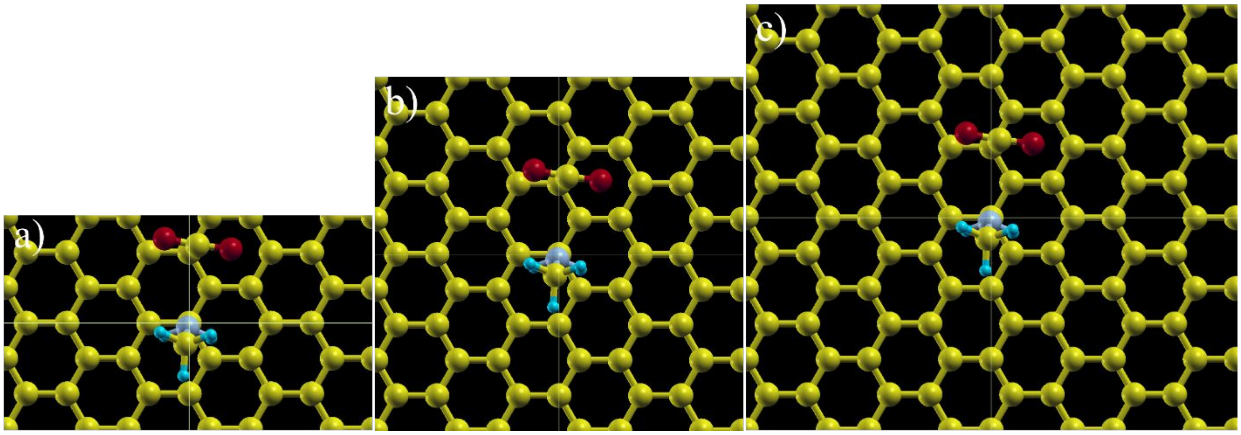

Figure S47. Single point configuration of CO_2 on GNH_2CH_3 in 36 (a), 60 (b), and 96 (c) C atom sheets using QE.

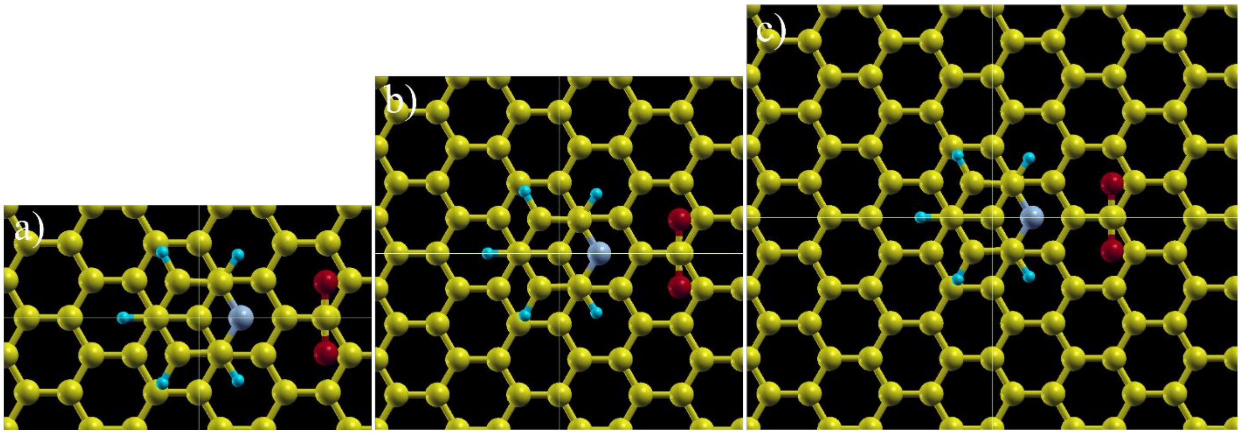

Figure S48. Single point configuration of CO_2 on GC_5H_5N in 36 (a), 60 (b), and 96 (c) C atom sheets using QE.

Van der Waals and Electrostatic Contributions in CO_2 E_{ads}

To understand our primary criterion of E_{ads}, we investigated the components contributing to E_{ads} in detail. More specifically, CO_2 E_{ads} can be decomposed into two major parts: electrostatic and van der Waals (vdW) interactions (see Table S9). The former results largely from the EN difference between the C atom of the CO_2 molecule and the N atom of the functional group, leading to dipole-induced dipole interactions. Thus, to satisfy our primary criteria, CO_2 must have favorable electrostatic interactions with the FMs and π-π dispersion interactions with the graphene surface, and they should add up to our target CO_2 E_{ads}. Methylamine and pyridine possess favorable electrostatic interactions with CO_2 (-0.15 eV and -0.15 eV), and their vdW interactions with graphene are similar (-0.18 and -0.16 eV, respectively). Interestingly, in both the pyridine and methylamine FG cases, the total, vdW, and ES energy contributions in CO_2+FM and CO_2+PG interactions are similar.

Table S9. Interaction energies (eV) of CO_2 with FMs and PG.

Materials	CO_2+FM			CO_2+PG		
	Total	vdW	ES	Total	vdW	ES
NH_2CH_3	-0.220	-0.074	-0.146	-0.178	-0.175	-0.003
C_5H_5N	-0.216	-0.069	-0.147	-0.171	-0.160	-0.011

Bader Charge Analysis

We investigated the Bader charge difference (BCD) and charge density difference (CDD) for a few N-containing FMs (see Figure 3 and Tables S10-S15). Interestingly, CO_2 is more stable interacting with amine in the inserted site over the top site as the former shows a higher amount of charge transfer compared to the latter. The other N-containing FMs also prefer to interact with CO_2 in the inserted sites. Amine and guanidine formed N-C bonds with CO_2 and show a higher BCD, CDD, and CO_2 E_{ads}, with a lower N-C distance. Although their CO_2 E_{ads} are the same, their BCD and CDD differ because of dissimilar initial Bader charge on N (6.13 e and 7.26 e) despite the final charges being similar (6.82 e and 6.92 e). The initial Bader charge of amine is lower due to the N-C bond formed with C of graphene. Since CO_2 physisorbs on aniline FG, it shows minimal BCD and CDD. Interestingly, pyridine and methylamine also show minimal BCD and CDD. In both cases, C (of CO_2) is favored to interact with N (of methylamine or pyridine) leading to distances of ~2.8 Å, which agrees with the BCD and CDD results.

Table S10. Bader Charge Difference (BCD (e)) for NH_2-T FG

Atom	BC w/ CO_2	BC	BCD
C	3.91	3.91	0.00
C	4.06	4.04	0.02
C	4.04	4.03	0.00
C	3.92	4.05	-0.13
C	4.06	3.91	0.15
C	4.01	4.03	-0.02
C	4.03	4.01	0.02
C	4.04	4.06	-0.02
C	3.94	3.94	0.00
C	4.01	4.00	0.00
C	4.02	4.02	0.00
C	4.06	4.06	0.00
C	3.91	3.91	0.00
C	3.99	3.99	0.00
C	4.01	4.02	-0.01
C	3.92	4.05	-0.13
C	4.06	3.91	0.15
C	4.00	4.00	0.00
C	4.00	4.00	0.00
C	4.04	4.06	-0.02
C	3.94	3.94	0.00
C	4.00	3.99	0.00
C	4.02	4.02	0.00

C	4.06	4.06	0.00
C	3.56	3.60	-0.04
C	4.06	4.04	0.02
C	4.04	4.03	0.00
C	3.95	3.95	0.01
C	4.03	4.03	0.00
C	4.01	4.03	-0.02
C	4.03	4.01	0.02
C	3.91	3.91	0.01
C	4.04	4.05	-0.01
C	4.01	4.00	0.00
C	4.02	4.02	0.00
C	3.99	3.99	0.00
N	6.16	6.13	0.02
H	0.60	0.59	0.01
H	0.58	0.60	-0.02
C	0.06	0.06	0.00
O	7.96	7.96	0.00
O	7.98	7.98	0.00

Table S11. BCD (e) for $C_6H_5NH_2$-I FG

Atom	BC w/ CO_2	BC	BCD
C	3.98	3.98	0.00
C	3.92	3.92	0.00
C	4.05	4.04	0.01
C	4.04	4.04	0.00
C	3.98	3.99	-0.01
C	3.92	3.92	0.00
C	4.05	4.05	0.00
C	4.03	4.03	0.00
C	3.98	3.99	0.00
C	3.92	3.91	0.00
C	4.05	4.05	0.00
C	4.03	4.03	0.00
C	4.02	4.03	0.00
C	3.92	3.93	0.00
C	4.05	4.05	0.00
C	4.03	4.03	0.00
C	4.03	4.03	0.00
C	3.92	3.92	0.00
C	4.05	4.06	-0.01

C	4.01	4.02	0.00
C	4.03	4.03	-0.01
C	3.92	3.92	0.00
C	4.06	4.05	0.00
C	4.02	4.02	0.00
C	4.02	4.02	0.00
C	3.91	3.92	0.00
C	4.03	4.03	0.00
C	4.03	4.03	0.00
C	4.04	4.03	0.01
C	3.92	3.92	0.00
C	4.03	4.04	0.00
C	4.02	4.02	0.01
C	4.03	4.03	0.00
C	3.91	3.91	0.00
C	4.03	4.03	0.00
C	4.01	4.01	0.00
N	6.74	6.76	-0.03
C	3.03	3.02	0.01
C	3.96	4.01	-0.05
C	3.97	4.01	-0.05
C	4.12	4.07	0.05
C	4.12	4.07	0.05
C	4.02	4.03	-0.01
H	0.97	0.97	0.00
H	0.97	0.97	0.00
H	0.96	0.96	-0.01
H	0.96	0.96	-0.01
H	0.98	0.96	0.02
H	0.60	0.59	0.01
H	0.60	0.59	0.01
C	0.07	0.07	0.00
O	7.97	7.96	0.01
O	7.97	7.97	0.01

Table S12. BCD (e) for C_5H_5N-I FG

Atom	BC w/ CO_2	BC	BCD
C	3.92	3.92	0.00
C	4.03	4.03	0.00
C	4.02	4.02	0.00
C	4.05	4.05	0.00

C	3.91	3.92	0.00
C	4.03	4.03	0.00
C	4.02	4.02	0.00
C	4.05	4.05	0.00
C	3.92	3.92	0.00
C	4.03	4.03	0.00
C	4.02	4.02	0.00
C	4.05	4.05	0.00
C	3.92	3.92	0.00
C	3.99	3.99	0.00
C	4.04	4.04	0.00
C	4.05	4.05	0.00
C	3.91	3.92	0.00
C	3.98	3.98	0.00
C	4.03	4.03	-0.01
C	4.05	4.05	0.00
C	3.92	3.92	0.00
C	3.99	3.98	0.00
C	4.04	4.04	0.00
C	4.05	4.05	0.00
C	3.91	3.91	0.00
C	4.03	4.03	0.00
C	4.02	4.02	0.00
C	4.03	4.03	0.00
C	3.91	3.91	0.01
C	4.03	4.03	0.00
C	4.02	4.02	0.00
C	4.03	4.03	0.00
C	3.91	3.91	0.00
C	4.03	4.03	0.00
C	4.02	4.02	0.00
C	4.03	4.03	0.00
N	7.65	7.67	-0.02
C	4.10	4.04	0.06
C	2.75	2.74	0.01
C	2.75	2.74	0.01
C	4.01	4.04	-0.03
C	4.01	4.04	-0.03
H	0.94	0.94	0.00
H	0.94	0.94	-0.01
H	0.93	0.94	-0.01

H	0.95	0.95	0.00
H	0.95	0.95	0.00
C	0.06	0.07	0.00
O	7.99	7.97	0.02
O	7.98	7.96	0.02

Table S13. BCD (e) for NH_2CH_3-I FG

Atom	BC w/ CO_2	BC	BCD
C	4.05	4.08	-0.03
C	4.05	4.01	0.03
C	4.04	4.04	0.00
C	3.91	3.91	0.00
C	4.04	4.07	-0.03
C	4.04	4.01	0.02
C	4.03	4.02	0.01
C	3.91	3.92	-0.02
C	4.04	4.07	-0.03
C	4.04	4.01	0.03
C	4.04	4.01	0.02
C	3.91	3.94	-0.03
C	4.06	4.06	0.00
C	4.01	4.01	0.00
C	4.00	4.00	0.00
C	3.92	3.91	0.02
C	4.06	4.07	-0.01
C	4.01	4.01	0.00
C	3.99	4.00	0.00
C	3.94	3.91	0.03
C	4.06	4.07	-0.01
C	4.01	4.00	0.00
C	3.99	4.00	-0.01
C	3.93	3.90	0.03
C	4.04	4.04	0.01
C	4.01	4.00	0.01
C	4.00	4.03	-0.03
C	3.91	3.91	0.00
C	4.04	4.04	0.00
C	4.01	4.01	0.00
C	4.00	4.04	-0.03
C	3.91	3.91	0.00
C	4.04	4.04	0.00

C	4.01	4.01	0.00
C	4.01	4.03	-0.03
C	3.92	3.91	0.01
C	3.64	3.66	-0.02
N	6.05	6.06	-0.01
H	1.03	1.01	0.02
H	0.65	0.65	0.00
H	0.64	0.64	0.00
H	0.98	0.99	-0.01
H	0.98	0.98	0.00
C	0.06	0.07	0.00
O	7.98	7.97	0.02
O	7.99	7.97	0.02

Table S14. BCD (e) for CH_5N_3-I FG

Atom	BC w/ CO_2	BC	BCD
C	3.91	4.07	-0.16
C	4.04	4.01	0.02
C	4.03	4.02	0.00
C	4.03	3.92	0.11
C	3.91	4.05	-0.14
C	4.04	4.02	0.02
C	4.01	4.02	0.00
C	4.04	3.92	0.12
C	3.91	4.05	-0.15
C	4.04	4.03	0.01
C	4.03	4.04	0.00
C	4.04	3.92	0.12
C	3.90	4.06	-0.16
C	4.02	4.03	-0.02
C	4.00	3.99	0.01
C	4.04	3.91	0.13
C	3.93	4.05	-0.11
C	4.01	4.03	-0.02
C	4.01	3.98	0.03
C	4.06	3.92	0.14
C	3.93	4.05	-0.12
C	4.01	4.03	-0.03
C	4.01	3.99	0.02
C	4.05	3.92	0.13
C	3.92	4.03	-0.12

C	3.99	4.00	-0.01
C	4.02	4.01	0.00
C	4.07	3.91	0.16
C	3.91	4.03	-0.11
C	4.00	4.02	-0.02
C	4.01	4.02	-0.01
C	4.07	3.92	0.15
C	3.91	4.03	-0.12
C	4.00	4.02	-0.03
C	4.02	4.04	-0.02
C	4.07	3.92	0.15
N	7.02	6.86	0.16
N	6.93	6.81	0.11
N	6.92	7.26	-0.33
C	0.06	0.06	0.00
H	0.46	0.57	-0.11
H	0.55	0.60	-0.05
H	0.56	0.59	-0.03
H	0.53	0.60	-0.07
H	0.55	0.64	-0.09
C	0.48	0.06	0.41
O	7.96	7.96	-0.01
O	7.98	7.98	0.00

Table S15. BCD (e) for NH_2-I FG

Atom	BC w/ CO_2	BC	BCD
C	4.03	3.91	0.12
C	4.03	4.04	-0.01
C	4.02	4.03	-0.01
C	3.91	4.05	-0.14
C	4.03	3.91	0.12
C	4.03	4.03	0.00
C	4.02	4.01	0.01
C	3.90	4.06	-0.16
C	4.04	3.94	0.10
C	4.03	4.00	0.03
C	4.03	4.02	0.01
C	3.90	4.06	-0.16
C	4.03	3.91	0.12
C	4.00	3.99	0.01
C	3.98	4.02	-0.04

C	3.92	4.05	-0.13
C	4.04	3.91	0.13
C	4.01	4.00	0.01
C	4.00	4.00	-0.01
C	3.92	4.06	-0.14
C	4.05	3.94	0.12
C	4.00	3.99	0.00
C	3.99	4.02	-0.03
C	3.89	4.06	-0.16
C	4.02	3.60	0.43
C	4.00	4.04	-0.05
C	3.97	4.03	-0.06
C	3.91	3.95	-0.04
C	4.03	4.03	0.00
C	4.01	4.03	-0.02
C	4.00	4.01	-0.02
C	3.90	3.91	-0.01
C	4.04	4.05	-0.01
C	3.99	4.00	-0.01
C	3.99	4.02	-0.03
C	3.89	3.99	-0.10
N	6.82	6.13	0.69
H	0.59	0.59	0.00
H	0.59	0.60	-0.01
C	0.06	0.07	0.00
O	7.70	7.97	-0.26
O	7.69	7.97	-0.28

References

(1) Hjorth Larsen, A.; Jørgen Mortensen, J.; Blomqvist, J.; Castelli, I. E.; Christensen, R.; Dułak, M.; Friis, J.; Groves, M. N.; Hammer, B.; Hargus, C.; Hermes, E. D.; Jennings, P. C.; Bjerre Jensen, P.; Kermode, J.; Kitchin, J. R.; Leonhard Kolsbjerg, E.; Kubal, J.; Kaasbjerg, K.; Lysgaard, S.; Bergmann Maronsson, J.; Maxson, T.; Olsen, T.; Pastewka, L.; Peterson, A.; Rostgaard, C.; Schiøtz, J.; Schütt, O.; Strange, M.; Thygesen, K. S.; Vegge, T.; Vilhelmsen, L.; Walter, M.; Zeng, Z.; Jacobsen, K. W. The Atomic Simulation Environment—a Python Library for Working with Atoms. *J. Phys. Condens. Matter* **2017**, *29* (27), 273002. https://doi.org/10.1088/1361-648X/aa680e.

(2) Giannozzi, P.; Baroni, S.; Bonini, N.; Calandra, M.; Car, R.; Cavazzoni, C.; Ceresoli, D.; Chiarotti, G. L.; Cococcioni, M.; Dabo, I.; Dal Corso, A.; de Gironcoli, S.; Fabris, S.; Fratesi, G.; Gebauer, R.; Gerstmann, U.; Gougoussis, C.; Kokalj, A.; Lazzeri, M.; Martin-Samos, L.; Marzari, N.; Mauri, F.; Mazzarello, R.; Paolini, S.; Pasquarello, A.; Paulatto, L.; Sbraccia, C.; Scandolo, S.; Sclauzero, G.; Seitsonen, A. P.; Smogunov, A.; Umari, P.; Wentzcovitch,

R. M. QUANTUM ESPRESSO: A Modular and Open-Source Software Project for Quantum Simulations of Materials. *J. Phys. Condens. Matter* **2009**, *21* (39), 395502. https://doi.org/10.1088/0953-8984/21/39/395502.

(3) Giannozzi, P.; Andreussi, O.; Brumme, T.; Bunau, O.; Buongiorno Nardelli, M.; Calandra, M.; Car, R.; Cavazzoni, C.; Ceresoli, D.; Cococcioni, M.; Colonna, N.; Carnimeo, I.; Dal Corso, A.; de Gironcoli, S.; Delugas, P.; DiStasio, R. A.; Ferretti, A.; Floris, A.; Fratesi, G.; Fugallo, G.; Gebauer, R.; Gerstmann, U.; Giustino, F.; Gorni, T.; Jia, J.; Kawamura, M.; Ko, H.-Y.; Kokalj, A.; Küçükbenli, E.; Lazzeri, M.; Marsili, M.; Marzari, N.; Mauri, F.; Nguyen, N. L.; Nguyen, H.-V.; Otero-de-la-Roza, A.; Paulatto, L.; Poncé, S.; Rocca, D.; Sabatini, R.; Santra, B.; Schlipf, M.; Seitsonen, A. P.; Smogunov, A.; Timrov, I.; Thonhauser, T.; Umari, P.; Vast, N.; Wu, X.; Baroni, S. Advanced Capabilities for Materials Modelling with Quantum ESPRESSO. *J. Phys. Condens. Matter* **2017**, *29* (46), 465901. https://doi.org/10.1088/1361-648X/aa8f79.

(4) Giannozzi, P.; Baseggio, O.; Bonfà, P.; Brunato, D.; Car, R.; Carnimeo, I.; Cavazzoni, C.; de Gironcoli, S.; Delugas, P.; Ferrari Ruffino, F.; Ferretti, A.; Marzari, N.; Timrov, I.; Urru, A.; Baroni, S. Q UANTUM ESPRESSO toward the Exascale. *J. Chem. Phys.* **2020**, *152* (15), 154105. https://doi.org/10.1063/5.0005082.

(5) Grimme, S. Semiempirical GGA-Type Density Functional Constructed with a Long-Range Dispersion Correction. *J. Comput. Chem.* **2006**, *27* (15), 1787–1799. https://doi.org/10.1002/jcc.20495.

(6) Barone, V.; Casarin, M.; Forrer, D.; Pavone, M.; Sambi, M.; Vittadini, A. Role and Effective Treatment of Dispersive Forces in Materials: Polyethylene and Graphite Crystals as Test Cases. *J. Comput. Chem.* **2009**, *30* (6), 934–939. https://doi.org/10.1002/jcc.21112.

(7) Blöchl, P. E. Projector Augmented-Wave Method. *Phys. Rev. B* **1994**, *50* (24), 17953–17979. https://doi.org/10.1103/PhysRevB.50.17953.

(8) van Setten, M. J.; Giantomassi, M.; Bousquet, E.; Verstraete, M. J.; Hamann, D. R.; Gonze, X.; Rignanese, G.-M. The PseudoDojo: Training and Grading a 85 Element Optimized Norm-Conserving Pseudopotential Table. *Comput. Phys. Commun.* **2018**, *226*, 39–54. https://doi.org/10.1016/j.cpc.2018.01.012.

(9) Henkelman, G.; Arnaldsson, A.; Jónsson, H. A Fast and Robust Algorithm for Bader Decomposition of Charge Density. *Comput. Mater. Sci.* **2006**, *36* (3), 354–360. https://doi.org/10.1016/j.commatsci.2005.04.010.

(10) Sanville, E.; Kenny, S. D.; Smith, R.; Henkelman, G. Improved Grid-Based Algorithm for Bader Charge Allocation. *J. Comput. Chem.* **2007**, *28* (5), 899–908. https://doi.org/10.1002/jcc.20575.

(11) Tang, W.; Sanville, E.; Henkelman, G. A Grid-Based Bader Analysis Algorithm without Lattice Bias. *J. Phys. Condens. Matter* **2009**, *21* (8), 084204. https://doi.org/10.1088/0953-8984/21/8/084204.

(12) Yu, M.; Trinkle, D. R. Accurate and Efficient Algorithm for Bader Charge Integration. *J. Chem. Phys.* **2011**, *134* (6), 064111. https://doi.org/10.1063/1.3553716.

(13) Momma, K.; Izumi, F. VESTA 3 for Three-Dimensional Visualization of Crystal, Volumetric and Morphology Data. *J. Appl. Crystallogr.* **2011**, *44*, 1272–1276. https://doi.org/10.1107/S0021889811038970.

(14) Patel, H. A.; Byun, J.; Yavuz, C. T. Carbon Dioxide Capture Adsorbents: Chemistry and Methods. *ChemSusChem* **2017**, *10* (7), 1303–1317. https://doi.org/10.1002/cssc.201601545.

(15) Koh, H. S.; Rana, M. K.; Hwang, J.; Siegel, D. J. Thermodynamic Screening of Metal-Substituted MOFs for Carbon Capture. *Phys. Chem. Chem. Phys.* **2013**, *15* (13), 4573. https://doi.org/10.1039/c3cp50622c.
(16) American Physical Society. *Direct Air Capture of CO2 with Chemicals*; A Technology Assessment for the APS Panel on Public Affairs; 2011. https://www.aps.org/policy/reports/assessments/upload/dac2011.pdf.
(17) Wang, C.; Fang, Y.; Duan, H.; Liang, G.; Li, W.; Chen, D.; Long, M. DFT Study of CO2 Adsorption Properties on Pristine, Vacancy and Doped Graphenes. *Solid State Commun.* **2021**, *337*, 114436. https://doi.org/10.1016/j.ssc.2021.114436.
(18) Wood, B. C.; Bhide, S. Y.; Dutta, D.; Kandagal, V. S.; Pathak, A. D.; Punnathanam, S. N.; Ayappa, K. G.; Narasimhan, S. Methane and Carbon Dioxide Adsorption on Edge-Functionalized Graphene: A Comparative DFT Study. *J. Chem. Phys.* **2012**, *137* (5), 054702. https://doi.org/10.1063/1.4736568.
(19) Zheng, Z.; Wang, H. Different Elements Doped Graphene Sensor for CO2 Greenhouse Gases Detection: The DFT Study. *Chem. Phys. Lett.* **2019**, *721*, 33–37. https://doi.org/10.1016/j.cplett.2019.02.024.
(20) Shokuhi Rad, A.; Pouralijan Foukolaei, V. Density Functional Study of Al-Doped Graphene Nanostructure towards Adsorption of CO, CO2 and H2O. *Synth. Met.* **2015**, *210*, 171–178. https://doi.org/10.1016/j.synthmet.2015.09.026.
(21) Cortés-Arriagada, D.; Villegas-Escobar, N.; Ortega, D. E. Fe-Doped Graphene Nanosheet as an Adsorption Platform of Harmful Gas Molecules (CO, CO2, SO2 and H2S), and the Co-Adsorption in O2 Environments. *Appl. Surf. Sci.* **2018**, *427*, 227–236. https://doi.org/10.1016/j.apsusc.2017.08.216.
(22) Mason, J. A.; Sumida, K.; Herm, Z. R.; Krishna, R.; Long, Jeffrey. R. Evaluating Metal–Organic Frameworks for Post-Combustion Carbon Dioxide Capture via Temperature Swing Adsorption. *Energy Environ. Sci.* **2011**, *4* (8), 3030. https://doi.org/10.1039/c1ee01720a.
(23) Fu, Y.; Yao, Y.; Forse, A. C.; Li, J.; Mochizuki, K.; Long, J. R.; Reimer, J. A.; De Paëpe, G.; Kong, X. Solvent-Derived Defects Suppress Adsorption in MOF-74. *Nat. Commun.* **2023**, *14* (1), 2386. https://doi.org/10.1038/s41467-023-38155-8.
(24) Caskey, S. R.; Wong-Foy, A. G.; Matzger, A. J. Dramatic Tuning of Carbon Dioxide Uptake via Metal Substitution in a Coordination Polymer with Cylindrical Pores. *J. Am. Chem. Soc.* **2008**, *130* (33), 10870–10871. https://doi.org/10.1021/ja8036096.
(25) Britt, D.; Furukawa, H.; Wang, B.; Glover, T. G.; Yaghi, O. M. Highly Efficient Separation of Carbon Dioxide by a Metal-Organic Framework Replete with Open Metal Sites. *Proc. Natl. Acad. Sci.* **2009**, *106* (49), 20637–20640. https://doi.org/10.1073/pnas.0909718106.
(26) Deng, H.; Grunder, S.; Cordova, K. E.; Valente, C.; Furukawa, H.; Hmadeh, M.; Gándara, F.; Whalley, A. C.; Liu, Z.; Asahina, S.; Kazumori, H.; O'Keeffe, M.; Terasaki, O.; Stoddart, J. F.; Yaghi, O. M. Large-Pore Apertures in a Series of Metal-Organic Frameworks. *Science* **2012**, *336* (6084), 1018–1023. https://doi.org/10.1126/science.1220131.
(27) McDonald, T. M.; Mason, J. A.; Kong, X.; Bloch, E. D.; Gygi, D.; Dani, A.; Crocellà, V.; Giordanino, F.; Odoh, S. O.; Drisdell, W. S.; Vlaisavljevich, B.; Dzubak, A. L.; Poloni, R.; Schnell, S. K.; Planas, N.; Lee, K.; Pascal, T.; Wan, L. F.; Prendergast, D.; Neaton, J. B.; Smit, B.; Kortright, J. B.; Gagliardi, L.; Bordiga, S.; Reimer, J. A.; Long, J. R. Cooperative Insertion of CO2 in Diamine-Appended Metal-Organic Frameworks. *Nature* **2015**, *519* (7543), 303–308. https://doi.org/10.1038/nature14327.

(28) Vlaisavljevich, B.; Huck, J.; Hulvey, Z.; Lee, K.; Mason, J. A.; Neaton, J. B.; Long, J. R.; Brown, C. M.; Alfè, D.; Michaelides, A.; Smit, B. Performance of van Der Waals Corrected Functionals for Guest Adsorption in the M_2 (Dobdc) Metal–Organic Frameworks. *J. Phys. Chem. A* **2017**, *121* (21), 4139–4151. https://doi.org/10.1021/acs.jpca.7b00076.
(29) Lee, J.-H.; Siegelman, R. L.; Maserati, L.; Rangel, T.; Helms, B. A.; Long, J. R.; Neaton, J. B. Enhancement of CO_2 Binding and Mechanical Properties upon Diamine Functionalization of M_2(Dobpdc) Metal–Organic Frameworks. *Chem. Sci.* **2018**, *9* (23), 5197–5206. https://doi.org/10.1039/C7SC05217K.
(30) Takeuchi, K.; Yamamoto, S.; Hamamoto, Y.; Shiozawa, Y.; Tashima, K.; Fukidome, H.; Koitaya, T.; Mukai, K.; Yoshimoto, S.; Suemitsu, M.; Morikawa, Y.; Yoshinobu, J.; Matsuda, I. Adsorption of CO_2 on Graphene: A Combined TPD, XPS, and vdW-DF Study. *J. Phys. Chem. C* **2017**, *121* (5), 2807–2814. https://doi.org/10.1021/acs.jpcc.6b11373.
(31) Lee, H. M.; Youn, I. S.; Saleh, M.; Lee, J. W.; Kim, K. S. Interactions of CO_2 with Various Functional Molecules. *Phys. Chem. Chem. Phys.* **2015**, *17* (16), 10925–10933. https://doi.org/10.1039/C5CP00673B.
(32) Bénard, P.; Chahine, R. Carbon Nanostructures for Hydrogen Storage. In *Solid-State Hydrogen Storage*; Elsevier, 2008; pp 261–287. https://doi.org/10.1533/9781845694944.3.261.
(33) Swenson, H.; Stadie, N. P. Langmuir's Theory of Adsorption: A Centennial Review. *Langmuir* **2019**, *35* (16), 5409–5426. https://doi.org/10.1021/acs.langmuir.9b00154.
(34) Ganesan, A.; Shaijumon, M. M. Activated Graphene-Derived Porous Carbon with Exceptional Gas Adsorption Properties. *Microporous Mesoporous Mater.* **2016**, *220*, 21–27. https://doi.org/10.1016/j.micromeso.2015.08.021.
(35) Xu, Q.; Wang, S.; Xue, L.; Shao, X.; Gao, P.; Lv, J.; Wang, Y.; Ma, Y. *Ab Initio* Electronic Structure Calculations Using a Real-Space Chebyshev-Filtered Subspace Iteration Method. *J. Phys. Condens. Matter* **2019**, *31* (45), 455901. https://doi.org/10.1088/1361-648X/ab2a63.
