# Supplementary material for: Carbon Capture: Theoretical Guidelines for Activated Carbon-Based CO2 Adsorption Material Evaluation
Source: J Phys Chem Lett. 2023 Nov 21;14(47):10693–9. doi: 10.1021/acs.jpclett.3c02711 (PMC10694831; doi:10.1021/acs.jpclett.3c02711)
Supplement: Supplementary file 2 — jz3c02711_si_002.pdf [file jz3c02711_si_002.pdf]

jz-2023-027115.R1

Name: Peer Review Information for "Carbon Capture: Theoretical Guidelines for Activated Carbon-based CO_2 Adsorption Material Evaluation"

First Round of Reviewer Comments

Reviewer: 1

Comments to the Author

The manuscript presented the theoretical guideline consisting of a hierarchy of crucial criteria that dictate the viability of AC-based materials for carbon capture. For this, the authors considered the optimal CO2 adsorption energy, thermal stability and material defects and selectivity and capacity. We found that methylamine and pyridine adsorb CO2 near the target. The various calculation data in this study is important in the future design of CO2 adsorbent. However, the authors should clarify some issues for the publication in JPCL.

1. What does FM stand for in the manuscript?

2. In Figure 2, the full name of FM, PG, DG, FG should be inserted.

3. How can the authors distinguish physisorption from chemisorption?

4. In graphene, authors considered the N, and O dopants to control electronegativity. How about B and P dopant? What is the reason why the authors did not consider this dopant?

5. The authors said that the FM must bind with PG in the chemisorption range for the stable operation of CO2 adsorption process. What is the guideline for chemisorption range ? This is based on calculation data or experimental data? The authors should discuss how the descriptor of thermal stability is chose.

6. In Fig 4(c), what is the meaning of CO2 adsorption energy? How this is related to defect sensitivity ? More Explanation is needed in manuscript.

7. It has been known that monovacancy can greatly affect the adsorption energy of CO2 over the pristine case. Why did authors consider this as the guidelines for defect sensitivity? Theoretical background for this one should be presented in manuscript.

8. The authors should show the optimal range of descriptors for representing CO2 adsorption energy, thermal stability and material defects and selectivity and capacity in detail. In addition, the authors should show the origin of optimal descriptor range in detail.

9. This study is related to the computational search scheme of graphene -based CO2 adsorbent. If other 2D materials is considered, the computational search scheme in this study can be applied? The authors need to discuss this point .

Author's Response to Peer Review Comments:

JPCL Editorial Comments and Our Responses

1. Please add postal codes to all author affiliations.
Thank you for bringing this to our attention. We included the postal codes in both the main paper and the SI on pages 1 and S1, respectively.

2. Please explain the # symbol after author names.
Thank you for bringing this to our attention. The first authors are indicated by the symbol #, which are Drew M. Glenna and Asmita Jana.

3. Please resize the TOC graphic per journal guidelines (2 in x 2 in).
Thank you for bringing this to our attention. We have changed the size of the TOC graphic on page 2 of the main paper.

4. Remove the section heading(s) throughout the body of the manuscript (you can leave Methods, Abstract, and TOC Graphic headings).
Thank you for bringing this to our attention and we have completed this.

5. A brief, nonsentence description of the actual contents of each supporting information file is required. This description should be labeled Supporting Information and should appear before the Acknowledgement and Reference sections.

Examples of sufficient and insufficient descriptions are as follows:

*Examples of sufficient descriptions: "Supporting Information: 1H NMR spectra for all compounds" or "Additional experimental details, materials, and methods, including photographs of experimental setup".

*Examples of insufficient descriptions: "Supporting Information: Figures S1-S3" or "Additional figures as mentioned in the text".

Thank you for bringing this to our attention. We have inserted this information on page 11.

"Supporting Information

Additional calculation details in figures and tables along with computational methods"

6. In both the main file and the supporting information, fix the style of all references to use JPCL formatting (check all references carefully). ***JPC Letters reference formatting requires that journal references should contain: () around numbers; author names; article title (titles entirely in title case or entirely in lower case); abbreviated journal title (italicized); year (bolded); volume

(italicized); and pages (first-last). Book references should contain author names; book title (in the same pattern); publisher; city; and year. Websites must include date of access.
Thank you for bringing this to our attention and we have completed this in both the main paper and the SI paper.

7. Please remove the Supporting Information file from the manuscript file. The SI file should only be uploaded as a separate Supporting Information for Publication file.
Thank you for bringing this to our attention and we have completed this.

8. Please number SI pages in the following format: "S1, S2…"
Thank you for bringing this to our attention and we have numbered the SI pages with S1, S2, etc.

9. Please include annotated version(s) of your revised publication file(s) with colored text or highlights indicating the revisions that you have made, and upload them as "Supporting Information for Review Only." Please also upload "clean" copies for publication. (No highlighting, annotations, or colored text.)
Thank you for bringing this to our attention and we have completed this.

JPCL Reviewer Comments and Our Responses

1. What does FM stand for in the manuscript?
   Thank you for pointing this out, this should not have slipped past us. FM stands for Functional Molecule. We have indicated this at its first occurrence, on page 4 of the main paper.

   "Next, for a lower EN difference (>0.9 $\sqrt{eV}$)[26] with CO_2, we moved to O-containing functional molecules (FMs) adsorbing in the basal plane on PG."

2. In Figure 2, the full name of FM, PG, DG, FG should be inserted.
   Thank you for pointing this out. Functional Molecule (FM), Pristine Graphene (PG), Doped Graphene (DG), and Functionalized Graphene (FG) are the terms. To be consistent, we have indicated all of these in the captions of figures 2, 3, and 4.

   "Figure 2. Dopants and functional molecules (FMs) screened in order from least to most stable CO_2 E_{ads} (a) and their corresponding CO_2 E_{ads} (b). The most stable CO_2 configurations on pristine graphene (PG), doped graphene (DG), and functionalized graphene (FG) are either top (T) or hollow (H) sites and T or inserted (I) sites, respectively. E_{ads} of the most stable sites are reported here whereas the comprehensive results of all sites can be found in Tables S1 and S3 and Figures S3-S8 and S20-S28."

   "Figure 3. Charge density difference (CDD) plots, Bader charge difference (BCD), CO_2 E_{ads}, and the distances between N (of functional molecule (FM)) and C (of CO_2) for graphene-derived materials (GDM) in top (T) and inserted (I) sites. BCD is reported for N (of FM), C (of CO_2), and both O (of CO_2) with top/left as O1 atom and right/bottom as O2 atom."

   "Figure 4. CO_2 E_{ads} (a) of most stable CO_2 configurations on pristine graphene (PG) and functionalized graphene (FG), (b) thermal stability by functional molecule (FM) E_{ads} on PG, (c) defect sensitivity by CO_2 E_{ads} on monovacancy graphene (MG), (d) selectivity by CO_2, N_2, O_2, and H_2O E_{ads}, and (e) coverage effects by CO_2 E_{ads} (single point calculations without ionically relaxing PG) on G36 (36 C atoms), G60 (60 C atoms), and G96 (96 C atoms) PG sheets."

3. How can the authors distinguish physisorption from chemisorption?
   Thank you for pointing this out and giving us the opportunity to explain further. We included a new section titled "Physisorption-Chemisorption Boundary on Graphene Materials" in the SI paper for more clarification on this topic (page S6).

   "From a physical chemistry perspective, physisorption typically corresponds to non-bonding interactions like van der Waals whereas chemisorption often involves a

significant charge transfer accompanied with formation of chemical bonds. More practically, the boundary between physisorption and chemisorption is commonly accepted to be in the range of -0.41 to -0.51 eV,[14] and we found it to be apt for our system. For instance, the CO_2 adsorption energy of methylamine functionalized graphene is right below the physisorption-chemisorption boundary (-0.367 eV), and we found that there was no N-C bond formed with a distance of 2.893 Å (see Table S3). In addition, CO_2 exhibited no Bader charge transfer with methylamine (see Table S13). On the other hand, imidazole functionalized graphene adsorbed CO_2 with an energy just above the physisorption-chemisorption boundary (-0.574 eV) and formed a N-C bond with a distance of 1.611 Å (see Table S3). Furthermore, CO_2 and imidazole showed Bader charge transfer with N (of $C_3N_2H_4$), C (of CO_2), O1 (of CO_2), and O2 (of CO_2) gaining -0.45, 0.21, 0.11, and 0.07 e, respectively. A similar CO_2 adsorption energy range (-0.41 to -0.78 eV) is commonly accepted for direct air capture applications.[15,16]"

4. In graphene, authors considered the N and O dopants to control electronegativity. How about B and P dopant? What is the reason why the authors did not consider this dopant?
   This is a great point and thank you for giving us the opportunity to explain why B and P were not considered as a dopant. They were not considered as a dopant due to the smaller differences in electronegativity with graphene and ultimately CO_2, in comparison to N and O. Additionally, it is very easy to include N and O as these elements are naturally more abundant than B and P. We included a sentence on page 4 for clarification.

   "In addition, this result ruled out B and P as dopants due to their smaller EN difference with C in graphene base material than dopants such as O and N. Therefore, we expect O and N to be better representatives of dopants for this system."

5. The authors said that the FM must bind with PG in the chemisorption range for the stable operation of CO_2 adsorption process. What is the guideline for chemisorption range? This is based on calculation data or experimental data? The authors should discuss how the descriptor of thermal stability is chosen.
   Thank you for giving us the opportunity to expand on this discussion point. The idea is that the material should be unchanged throughout the CO_2 desorption process, which in our case is heating the material to release CO_2. If the material does not change during the heating process, then the material is thermally stable. Since our target CO_2 adsorption energy is near the physisorption-chemisorption boundary, the material must be sufficiently above the chemisorption boundary and therefore well into the chemisorption range. If the functional molecule adsorbs on graphene in the chemisorption range (< -0.41 eV), then the material is thermally stable. All of our studies were computational and this is also a computational guideline. We included a definition of thermal stability on pages 7 and 8 for additional clarification.

   "We define thermal stability as the ability of a material to stay unaltered during the CO_2 desorption process." For thermal stability, the FM must bind with PG in the

chemisorption range to eliminate their removal post CO_2 desorption. "While a general guideline is hard to propose, a higher E_{ads} (in the chemisorption range) of the FM on PG compared to CO_2 E_{ads} on the corresponding FG is needed to ensure thermal stability."

6. In Fig 4(c), what is the meaning of CO_2 adsorption energy? How is this related to defect sensitivity? More explanation is needed in the manuscript.
   Thank you for giving us the opportunity to explain this criterion further. The CO_2 adsorption energy in Fig 4(c) corresponds to the adsorption energy of CO_2 on a structure that includes a monovacancy defect. We meant defect sensitivity in the sense of how CO_2 interacts or adsorbs on the material in the presence of defects in comparison to the case where no defects are included. We included a definition along with the reason we chose monovacancy defects in particular on page 8.

   "We define a defect insensitive material as a desirable material whose CO_2 E_{ads} does not change when defects are introduced. In other words, the CO_2 E_{ads} of the pristine material and the material with defects are the same. Thus, to ensure the properties of the capture material are unaffected by the presence of defects, CO_2 E_{ads} must be invariant between PG and defect graphene. We considered monovacancy graphene (MG) defects as they alter CO_2 E_{ads} more than any other graphene defect (like the Stone-Wales defect)[19,27] due to the dangling C bonds interacting more strongly with CO_2 (see Figure 4c, Table S5, and Figure S31)."

7. It has been known that monovacancy can greatly affect the adsorption energy of CO_2 over the pristine case. Why did authors consider this as the guidelines for defect sensitivity? Theoretical background for this one should be presented in manuscript.
   Thank you for allowing us to expand on this statement. We considered monovacancy graphene defects as they alter CO_2 E_{ads} more than any other graphene defect. We expanded on this statement on page 8.

   "We considered monovacancy graphene (MG) defects as they alter CO_2 E_{ads} more than any other graphene defect (like the Stone-Wales defect)[19,27] due to the dangling C bonds interacting more strongly with CO_2 (see Figure 4c, Table S5, and Figure S31)."

   We pointed the defect results in the SI tables and figures into the main paper on page 8.

   "Methylamine adsorbs onto MG near the lower chemisorption threshold (see Table S4 and Figure S29) while adsorbing CO_2 near our target CO_2 E_{ads} (see Figure 4c, Table S5, and Figure S32) and is therefore a defect insensitive material. However, pyridine forms two C-C bonds with MG and is much more stable than on PG (see Table S4 and Figure S30), but pyridine is near perpendicular to MG resulting in a less stable CO_2 E_{ads} when compared to pyridine FG (see Figure 4c, Table S5, and Figure S33)."

8. The authors should show the optimal range of descriptors for representing CO_2 adsorption energy, thermal stability and material defects and selectivity and capacity in detail. In addition, the authors should show the origin of optimal descriptor range in detail.

Thank you for pointing this out and allowing us to expand on our criteria for carbon capture evaluation. We included the following to serve as a summary of the descriptors and their range in page 10-11.

"To ensure optimum performance, we opted for a target CO_2 E_{ads} of -0.41 eV. Moreover, a good candidate material must also be thermally stable during the CO_2 desorption process. Only those FMs which adsorbed on graphene with a higher energy (< -0.41 eV) compared to the E_{ads} of CO_2 on graphene functionalized with that group passed this criterion of thermal stability. Since defects are unavoidable, it is imperative that the CO_2 E_{ads} remain unchanged in the presence of defects. Additionally, a good candidate material must also preferentially adsorb CO_2 over other gases. In other words, the selectivity criterion requires the CO_2 E_{ads} to be higher than the rest. Finally, the CO_2 E_{ads} can also vary with the density or coverage of the FMs, and for optimum capacity, a FM that supports the target CO_2 E_{ads} with high coverage should be chosen."

The origin of the target CO_2 adsorption energy comes from choosing an adsorption energy that is between physisorption and chemisorption. This is because "Optimum performance necessitates an ideal range of E_{ads} where CO_2 is bound neither too strongly for ease in desorption nor too weakly for good selectivity" as stated in page 2 in the manuscript. We also included some discussion on the boundary of physisorption and chemisorption in the SI (page S6).

"From a physical chemistry perspective, physisorption typically corresponds to non-bonding interactions like van der Waals whereas chemisorption often involves a significant charge transfer accompanied with formation of chemical bonds. More practically, the boundary between physisorption and chemisorption is commonly accepted to be in the range of -0.41 to -0.51 eV,[14] and we found it to be apt for our system. For instance, the CO_2 adsorption energy of methylamine functionalized graphene is right below the physisorption-chemisorption boundary (-0.367 eV), and we found that there was no N-C bond formed with a distance of 2.893 Å (see Table S3). In addition, CO_2 exhibited no Bader charge transfer with methylamine (see Table S13). On the other hand, imidazole functionalized graphene adsorbed CO_2 with an energy just above the physisorption-chemisorption boundary (-0.574 eV) and formed a N-C bond with a distance of 1.611 Å (see Table S3). Furthermore, CO_2 and imidazole showed Bader charge transfer with N (of $C_3N_2H_4$), C (of CO_2), O1 (of CO_2), and O2 (of CO_2) gaining -0.45, 0.21, 0.11, and 0.07 e, respectively. A similar CO_2 adsorption energy range (-0.41 to -0.78 eV) is commonly accepted for direct air capture applications.[15,16]"

Other rationales for the choice of -0.41 eV as the target CO_2 adsorption energy comes from parasitic energy studies in COFs and a DFT calculation on Mg-MOF-74, which is taken as a standard for CO_2 adsorption. The following explanation in the manuscript can be found in page 4.

"The less stable boundary of this threshold range is consistent with our target CO_2 E_{ads}, which is motivated by the parasitic energy metric,[23] based on monoethanolamine, an industry standard,[24] used to screen covalent organic frameworks (COFs) for carbon capture in Deeg et al.[24] Parasitic energy contains the energy required to separate CO_2 from flue gas ($Q_{separation}$), which should be minimized to reduce energy losses resulting in a CO_2 heat of adsorption threshold of -0.46 eV.[24] Further, the best COFs are comparable to Mg-MOF-74 (metal organic framework). A DFT calculation of CO_2 E_{ads} on Mg-MOF-74 is -0.41 eV (see Figure S2), which is assigned as our target CO_2 E_{ads}."

9. This study is related to the computational search scheme of graphene-based CO_2 adsorbent. If other 2D materials is considered, the computational search scheme in this study can be applied? The authors need to discuss this point.
Thank you for pointing this out to us and this is an excellent addition to our paper. We hypothesize that this theoretical guideline can be easily adapted for evaluating other 2D materials for CO_2 capture. The list of criteria devised for CO_2 capture that include optimum adsorption energy, thermal stability, defect insensitivity, high selectivity, and capacity is material agnostic. More specifically, the operational ranges for the secondary and ternary criteria are quantified relative to the primary CO_2 adsorption energy, and thus those criteria remain unchanged. However, CO_2 adsorption depends on the specifics of how CO_2 interacts with the solid-sorber material, which in turn will influence the target adsorption energy. We included a statement touching on this in our conclusion on page 11.

"Furthermore, this theoretical guideline can be generalized to different carbon capture materials, like other 2D materials (MoS_2, WSe_2, CrO_2, CrS_2, VO_2, VS_2, h-BN, $NbSe_2$, etc.), since the list of criteria devised for CO_2 capture that include optimum E_{ads}, thermal stability, defect insensitivity, high selectivity, and capacity is material agnostic. The CO_2 E_{ads}, the primary parameter that all the other criteria are dependent on, in turn relies on the knowledge of fundamental interactions between CO_2 and the solid-sorber of interest. Such studies will play a critical role in the future designs of CO_2 capture solutions."
